# Field-Grown Grapevine Berries Use Carotenoids and the Associated Xanthophyll Cycles to Acclimate to UV Exposure Differentially in High and Low Light (Shade) Conditions

**DOI:** 10.3389/fpls.2016.00786

**Published:** 2016-06-10

**Authors:** Chandré Joubert, Philip R. Young, Hans A. Eyéghé-Bickong, Melané A. Vivier

**Affiliations:** ^1^Department of Viticulture and Oenology, Stellenbosch UniversityStellenbosch, South Africa; ^2^Institute for Wine Biotechnology, Stellenbosch UniversityStellenbosch, South Africa; ^3^Institute for Grape and Wine Sciences, Stellenbosch UniversityStellenbosch, South Africa

**Keywords:** UVB radiation, solar radiation, climate change adaptation, acclimation, berry development

## Abstract

Light quantity and quality modulate grapevine development and influence berry metabolic processes. Here we studied light as an information signal for developing and ripening grape berries. A *Vitis vinifera* Sauvignon Blanc field experiment was used to identify the impacts of UVB on core metabolic processes in the berries under both high light (HL) and low light (LL) microclimates. The primary objective was therefore to identify UVB-specific responses on berry processes and metabolites and distinguish them from those responses elicited by variations in light incidence. Canopy manipulation at the bunch zone via early leaf removal, combined with UVB-excluding acrylic sheets installed over the bunch zones resulted in four bunch microclimates: (1) HL (control); (2) LL (control); (3) HL with UVB attenuation and (4) LL with UVB attenuation. Metabolite profiles of three berry developmental stages showed predictable changes to known UV-responsive compound classes in a typical UV acclimation (versus UV damage) response. Interestingly, the berries employed carotenoids and the associated xanthophyll cycles to acclimate to UV exposure and the berry responses differed between HL and LL conditions, particularly in the developmental stages where berries are still photosynthetically active. The developmental stage of the berries was an important factor to consider in interpreting the data. The green berries responded to the different exposure and/or UVB attenuation signals with metabolites that indicate that the berries actively managed its metabolism in relation to the exposure levels, displaying metabolic plasticity in the photosynthesis-related metabolites. Core processes such as photosynthesis, photo-inhibition and acclimation were maintained by differentially modulating metabolites under the four treatments. Ripe berries also responded metabolically to the light quality and quantity, but mostly formed compounds (volatiles and polyphenols) that have direct antioxidant and/or “sunscreening” abilities. The data presented for the green berries and those for the ripe berries conform to what is known for UVB and/or light stress in young, active leaves and older, senescing tissues respectively and provide scope for further evaluation of the sink/source status of fruits in relation to photosignalling and/or stress management.

## Introduction

Plants not only use solar light to drive photosynthesis and energy production, they also use it as a source of information about their environment. New information regarding the impact of the different spectral components of solar light (visible, UVA and UVB) are emerging, causing paradigm shifts with regards to the interpretation of existing and new results, the methods of experimentation, as well as the development of hypothesis and models to understand the intricate modulating effects versus the stress responses evoked by light components (Hideg et al., 2013). In the study of UV effects, it is now established that under ecological/field conditions, plants rarely display the classical UV damage phenotypes that have been established. Instead, a more complex picture is emerging showing that low ecologically relevant doses of UV are used by plants to acclimate and to modulate core processes to remain productive and thriving (Hideg et al., 2013; Li et al., 2013).

UVB (280–315 nm) is an intrinsic part of solar radiation and is no longer considered a generic abiotic stress factor, but has been demonstrated to be a specific modulator. This is supported by the fact that UVB radiation is required for photomorphogenic responses (including acclimation) and is essential in the formation of the UVB photoreceptor, UVR8. In the absence of UVB radiation, UVR8 occurs as an inactive dimer (homo-dimers connected by salt bridges). UVB radiation causes a rapid accumulation of the active monomeric form of UVR8 in the nucleus, where the protein directly binds chromatin via histones. UVB radiation neutralizes the salt bridges (connecting the UVR8 homodimers) resulting in the release of the active UVR8 monomers. The UVR8 monomers subsequently conjugate with COP1, and this UVR8-COP1 conjugate activates the transcription of HY5. HY5, a bZIP transcription factor, subsequently regulates numerous light-responsive genes (>100 in *Arabidopsis*) involved in photomorphogenesis (Favory et al., 2009). In the absence of UVB radiation, UVR8 monomer dimerization is catalyzed by WD40-repeat proteins RUP1 and RUP2 (in *Arabidopsis thaliana*). Photomorphogenic responses to UVB radiation in leaves include reduced leaf expansion, increased leaf thickness, accumulation of phenolic compounds (predominantly flavonoids) and cuticular waxes (Tilbrook et al., 2013). These responses are comprehensively described for a number of plant species and specifically in photosynthetic organs (predominantly leaves), but data from fruit acclimation suggest that fruit in the early developmental stages, when chloroplasts are still functionally photosynthesizing, react in much the same way as leaves (via photo-protective mechanisms with the purpose of maintaining photosynthesis) (Blanke and Lenz, 1989).

Grapes are fleshy fruits grown in temperate areas of the world where a large proportion of similar cultivated varieties are produced under vastly different environmental conditions. The different climatic zones in viticultural production areas have been extensively characterized, particularly considering the potential impacts of climate change on berry metabolism and consequent quality. The responses of field-grown plants (including grapevine) to biotic and abiotic stress are complex. Plants are typically exposed to multiple stresses and their responses are dynamic and overlapping and are classified as elastic (reversible) or plastic (irreversible) responses (reviewed in Cramer et al., 2011). Changes in the environment necessitate the alteration of the plant’s phenotype in order to adapt to external environmental factors. This is referred to as phenotypic plasticity and is deemed the foremost method employed by plants to cope with environmental changes. *Vitis vinifera* has been shown to display phenotypic plasticity under these diverse conditions, particularly evidenced in berry transcripts and metabolites (Dal Santo et al., 2013; Young et al., 2016).

The limited research on grapevine berries and UV exposure in natural settings have shown that cultivated varieties are relatively well adapted to ambient UV exposure and typically show acclimation and not UV stress responses. Similarly, studies on other fruits and crops have revealed that acclimation responses to natural UVB levels involve the production of UVB absorbing flavonoids and phenolics. It has been shown that in some instances these compounds can act as UVB screens directly (Kolb et al., 2003), whereas in other occasions and/or locations, the inherent antioxidant capacity of the same compounds rather contributes to acclimation responses (Carbonell-Bejerano et al., 2014). The current understanding of UV effects on grapevine organs conforms to what is known for other species, i.e., with regards to the regulating aspects of UV stimuli, the phenylpropanoid pathway has been strongly linked to UV exposure. The observation that the attenuation of UVB reduces the accumulation of UVB absorbing compounds is not unique to grapevine and has been shown in a number of other fruits, including: apple (Arakawa et al., 1985; Ubi et al., 2006), tomato (Calvenzani et al., 2010) and blackcurrant (Huyskens-Keil et al., 2012).

Several studies have focused on UV effects on grapevine berries (Gregan et al., 2012; Gil et al., 2013; Carbonell-Bejerano et al., 2014), with some reports on vegetative and/or whole plant physiological performance (Pontin et al., 2010; Martínez-Lüscher et al., 2013). It has been demonstrated that the flavonoid biosynthetic pathway is transcriptionally regulated by UVB radiation in the skin of berries (Downey et al., 2004; Carbonell-Bejerano et al., 2014). Interestingly, a recent study on Sauvignon Blanc berries under different light and UV regimes lends support to the notion that in grapevine berries the biosynthesis of flavonols are increased through the classical low fluence UVB response pathway (Tian et al., 2015). Moreover, in the ripe berry stages putative terpenoid biosynthetic genes encoding for linalool and eucalyptol were upregulated in *V. vinifera* L. cv. Tempranillo in response to UVB radiation (Carbonell-Bejerano et al., 2014). Although these studies have identified possible regulatory genes and stress pathways that could be involved in UVB stress/acclimation, significant gaps still exist in our understanding of the mechanisms (and biological drivers) behind the observed responses. Additional motivation exists to clarify the effects of UV and general solar radiation on berry (and fruits in general) composition, since it is accepted to impact berry and wine quality.

The hypothesis of this study was that under field conditions high/low photosynthetically active radiation (PAR) and high/low UV exposures contribute in different ways to the response of berries to solar exposure. Our primarily objective was to distinguish between UV and PAR-specific responses on berry metabolites. To this end we evaluated Sauvignon Blanc berries in a high-altitude (model/highly characterized) vineyard where an experimental system to study berry metabolism under low and high (PAR) light exposure in the bunch zones was validated previously (Young et al., 2016). It was reported that specific metabolites responded to increased solar exposure [PAR + UV = High Light (HL)] in a metabolically plastic pattern in a likely process of antioxidant homeostasis, involving different metabolites depending on the developmental stage of the berries and when compared to the low light (LL) control. This characterized HL and LL experimental system provided an excellent opportunity to evaluate the specific responses and/or contribution of UV exposure to the metabolic responses. UV exclusion sheets were used to attenuate UVB light exposure (>99% reduction) on the berries under these two light regimes. In the first two seasons of the study, we found a strong light (PAR) and UV effect on specific berry carotenoid pigments, prompting a comprehensive analysis of the carotenoids and their derivatives (norisoprenoids) in subsequent seasons. Apart from two earlier studies by Schultz et al. (1998) (reporting total carotenoids and zeaxanthin in Riesling) and Steel and Keller (2000) (β-carotene and lutein in Cabernet Sauvignon), the impact of UV exposure on the photosynthetic pigments in berries is still relatively poorly described (compared to e.g., polyphenolics in red cultivars). Our results extend the current understanding of UV impacts in grapevine fruits (and fruits in general) by showing that specific carotenoids involved in photoprotection are responsive to levels of solar radiation (exposure), but that the UVB component in this light signal is required for the typical photo-protective response linked to the violaxanthin cycle under HL, as well as the accumulation of lutein epoxide under LL conditions. The ripe berry stages in particular displayed the accumulation of volatile compounds, but the profiles and levels depended on the specific level of exposure and UVB presence/absence. The results are discussed within the context of fruit metabolism in reaction to light as a source of information to modulate core processes.

## Materials and Methods

### Vineyard Treatment, Experimental Design, and Berry Sampling

A model *Vitis vinifera* L. cv. Sauvignon Blanc vineyard established in a commercial vineyard situated in the Elgin area of South Africa was used for the experiment. The vines were orientated in a north-west, south-east row direction and trained on a vertical shoot positioned (VSP) trellis system. Spur pruning to two buds was employed during winter and diligent canopy management occurred throughout the growing season. No water constraints were noted due to the high moisture content of the deep shale soils, as was confirmed by stem water potential measurements in the same vineyard and reported in Young et al. (2016).

The experimental plot included three rows from which 16 panels were selected. Two controls and two treatments were applied randomly over the 16 panels with each control/treatment being repeated four times. Each panel consisted of four consecutive vines and represented a single biological repeat (**Supplementary Figure S1** shows a diagram of the plot layout as well as images of the treatments).

Canopy manipulation via basal leaf and lateral shoot removal in the bunch zone (30–40 cm above the cordon) resulted in an altered exposure of the grape berries to light, thereby creating two distinctive bunch microclimates (with reference to exposure). This was done only on the East-facing side of the canopy, namely the side which was exposed to sunlight in the morning. A full characterization of the leaf removal treatment was recently reported in Young et al. (2016) that delivered a validated exposed versus a shaded bunch microclimate. UV light manipulation was achieved by installing UV-excluding acrylic sheets (Perspex^®^ South Africa) over the bunch zone. The following four scenarios were therefore created in the vineyard: (1) complete leaf and lateral shoot removal in the bunch zone (30–40 cm above the cordon) on the morning side of the canopy (East side), generating the High Light control (HLcontrol); (2) a similar scenario to the first with the addition of a UVB excluding acrylic sheet installed over the bunch zone, generating the High Light-UVB (HL-UVB) treatment; (3) no leaf or lateral shoot removal, constituting a fully shaded situation, generating the Low Light control (LLcontrol); (4) and a similar scenario to the third with the addition of a UVB excluding sheet over the bunch zone, generating the Low Light-UVB (LL-UVB) treatment.

Leaf and lateral removal as well as the installation of the UV-excluding sheets were carried out when the berries reached peppercorn size according to the Eichorn and Lorenz (EL) system (EL 29) (Eichhorn and Lorenz, 1977). Sampling of the berries occurred at pea-sized berries (EL31), véraison (EL35), and ripe (corresponding to the harvest date; EL38) to yield samples that covered the full growing and ripening season. The stages corresponded to 26, 67, and 107 DAA (days after anthesis) in the 2011/2012 season and 25, 66, and 96 DAA in the 2014/2015 season. Berry sampling was carried out at each of the phenological stages on a per panel basis and therefore comprised of four biological repeats per treatment. Each sample consisted of 48–50 berries. Representative bunches on the exposed side (east-facing) of the canopy were selected from which to sample. Care was taken to select only berries from the exposed side of the selected bunches. Samples were frozen immediately after being picked in the field using liquid nitrogen and then transported to the laboratory. The seeds were removed and the remaining tissue milled in liquid nitrogen, after which they were stored at -80°C until analyzed.

The trial was conducted over multiple seasons (2011/2012; 2013/2014; 2014/2015), but metabolite profiling mainly occurred in the first and last season and will be presented in the results section.

### Climatic Measurements

Climatic monitoring (meso-and micro-) occurred in the vineyard to quantify the main abiotic factors which could influence grapevine growth and development in response to the treatments. Various loggers and sensors were placed in the vineyard to measure climatic variables.

Temperature was measured at the mesoclimatic level via Tinytag^®^ loggers (TinyTag Plus 2 – TGP-4500., Gemini Data Loggers (UK) Ltd., Chichester, United Kingdom) installed above the canopy. Similar loggers were placed within the canopy to measure temperature on a microclimatic scale. Bunch temperatures were monitored using dual channel temperature data loggers to which two thermistor flying lead probes were attached (TinyTag Plus 2 – TGP-4520). These probes were positioned within selected bunches from each of the controls and treatments. With regard to light measurements, both solar radiation (including PAR) and UV radiation were monitored. Solar radiation sensors (Vantage Pro2^TM^ solar radiation sensors Davis Instruments, Hayward, CA, USA) were also installed inside and outside the canopy. The outer unit measured the ambient solar radiation while the internal sensors measured the solar radiation which penetrated the canopy and reached the bunch zone. A solar sensor was placed in the bunch zone of each of the four light environments to determine the degree of light penetration in each case. UV radiation was measured using sensors (Apogee SU-100 UV sensors. Apogee Instruments Inc., Logan, UT, USA) which were positioned similarly to the solar radiation sensors; one externally to measure ambient UV and one placed in the bunch zone of each created light environment. The solar and UV sensors were attached to two loggers (DataTaker DT82E data logger, Thermo Fisher Scientific Australia Pty Ltd, Melbourne, VIC, Australia) which recorded measurements throughout berry development.

### Analysis of Major Sugars and Organic Acid Concentrations

The major sugars and organic acids of the berries were extracted and analyzed using HPLC as described in Eyéghé-Bickong et al. (2012).

### Analysis of Photosynthetic Pigment Concentrations

The carotenoids and chlorophylls of the berries were extracted and analyzed using UPLC as described in Lashbrooke et al. (2010) and Young et al. (2016) respectively. The de-epoxidation state (DEPS) of the xanthophylls were calculated as (zeaxanthin + 0.5antheraxanthin)/(violaxanthin + zeaxanthin + antheraxanthin) as described in Thayer and Björkman (1990).

### Analysis of Volatile Aroma Compounds

All authentic standards for volatile analysis were purchased from Sigma Aldrich (Steinheim, Germany): 6-methyl-6-heptan-2-one, trans-2-hexanol, 2-octenal, d-anisol, trans-2-heptanal, geralnylacetone, eucalyptol, limonene, trans-linalool-oxide, *cis*-linalool-oxide, linalool, 4-terpeneol, citronellol, nerol, geraniol, β-damascenone, α-ionone, β-ionone and pseudo-ionone, β-damascone and α-terpineol. Tartaric acid, ascorbic acid, sodium chloride (NaCl), sodium azide (NaN_3_) and methanol were also acquired from Sigma Aldrich. For extraction of volatiles from grape berry tissue, approximately 1 g of ground, frozen tissue was weighed into a 20 mL GC vial and 2 mL of tartaric acid buffer (2 g.L^-1^ tartrate, 2.1 g.L^-1^ ascorbic acid and 0.8 mg.L^-1^ L^-1^ sodium azide; pH 3) was added to each vial. Volatiles were extracted by head space (HS) solid phase microextraction (SPME) using a 50/30 μm divinylbenzene/carboxen/polydimethylsiloxane (DVB/CAR/PDMS) fiber (2 cm gray fiber from Supelco, Bellefonte, PA, USA) (Barros et al., 2012). Prior to use, the fiber was conditioned at 270°C for 60 min in the GC injection port according to the manufacturer’s specifications.

The samples were equilibrated at 60°C for 5 min in a heating chamber (with constant agitation at 250 rpm). After equilibration, the SPME fiber was inserted through the vial septa and exposed to the sample at 60°C for 30 min with constant agitation at 250 rpm. The bound analytes were thermally desorbed from the fiber in the GC injection port. After desorption, the fiber was maintained for 20 min in the injection port for cleaning in order to prevent potential carryover between samples.

GC analysis was carried out on an Agilent 6890N gas chromatograph (Agilent, Palo Alto, CA, USA) system coupled to a CTC CombiPal Analytics auto-sampler and an Agilent 5975B inert XL EI/CI MSD mass spectrometer detector through a transfer line. Analysis was done using a Zebron 7HG-G009-11 capillary column (30 m × 250 μm ID, 0.25 μm). Desorption of analytes from the SPME fiber was performed in the injection port at 250°C by pulsed splitless mode for 1 min. The purge flow was 30 mL.min^-1^ (for 2 min). The column operating head pressure was raised from 111 kPa to obtain a pulse pressure of 300 kPa for 1 min. Helium was used as carrier gas with a constant flow rate of 1 mL.min^-1^. The oven parameters were as follows: initial temperature of 40°C (2 min), a linear increase to a final temperature of 240°C (at a rate of 10°C.min^-1^), and the temperature was held at 240°C for a final 2 min. The total run time was 28 min. The transfer line temperature was maintained at 250°C. The MS detector was operated in scan and selected ion monitoring (SIM) modes. The scan parameters were set ranging from 35 to 350 m/z. The dwell time for each ion in a group was set to 100 ms. The software used was MSD ChemStation (G1701-90057, Agilent).

For quantification, external standard calibration was done by plotting standard curves using the ratio of the peak area of each authentic standard relative to that of the internal standard, versus the standard concentration (see **Supplementary Table S1** for calibration parameters). Volatiles in samples were identified according to their elution times and masses compared to those of the respective authentic standards and quantified using the calibration parameters. Compounds without available authentic standard were identified by matching their mass spectrum with the Wiley 275 mass spectral library (Wiley, New York, NY, USA) and quantified. The resulted concentrations in μg/L were then divided by the berry fresh weight and multiplied by the sample volume (2 mL) to obtain the content (in ng/g FW). The selected ions used for the integration of peak areas of the respective compounds of interest, their retention time on the Zebron column, and quantifier molecules are summarized in **Supplementary Table S2**.

### Analysis of Polyphenolics

Total polyphenolic acids were analyzed by HPLC on an Agilent 1200 at the Oxidative Stress Research Centre, Cape Peninsula University of Technology, Bellville, South Africa.

### Statistical Analysis

The resulting datasets were evaluated statistically, and were subjected to multivariate data analyses to integrate the different data layers. Microsoft Excel and Statistica (version 12) were utilized for standard statistical analysis. The responses of the various compounds to the individual treatments were tested for significance using a pairwise *t*-test. Testing was conducted on a “per developmental stage” basis. The contrasts examined were separated into HL and LL comparisons, thereby allowing for the examination of the effects of UV in a HL environment [HLcontrol (HL + ambient UV) versus HL-UVB] as well as a LL environment [LLcontrol (LL + ambient UV) versus LL-UVB]. Analysis of variance (ANOVA) was conducted on those pairwise contrasts with a *p*-value of <0.05. Linear models were fitted to the contrasts showing significant variation in order to visualize the actual concentrations of the relevant compounds during berry development. Similar testing was conducted on the climate data to identify the main treatment effect(s).

Furthermore, a repeated measures ANOVA was conducted on the data in order to rank the significance of each compound in response to the three main experimental factors (i.e., development, light exposure and UVB radiation) individually and in combination. A repeated measures ANOVA was used to test for potential cause-effect relationships between the measured compounds and the main experimental factors. The results of the ANOVA are reported as *F*-values. The higher the *F*-value is, the lower the *p*-value, and the greater the significance will be. Fisher LSD *Post Hoc* tests were used to confirm which compounds reacted statistically significantly to the specified factors (adjusted *p*-value, *q*-value).

Multivariate data analysis was conducted using SIMCA (version 12.0.3.0 from MKS Data Analytics and Solutions). The data was analyzed using orthogonal partial least squares – discriminant analysis (OPLS-DA). These models are used to relate the data matrix (X, the measured metabolites) to a specified qualitative vector (Y, class, e.g., developmental stage, exposure or UV). The use of supervised OPLS-DA models assisted in the visualization of the complex datasets which consisted of multiple variables and helped to identify putative correlations within the dataset. The score plots are related to the individual observations which are grouped into similar patterns. The corresponding loading plots are used to relate the observed patterns in the OPLS-DA to the measured variables. Coefficient plots are displayed here in lieu of loading plots as they give an indication of direction. The X-variables are scaled and centered and the regression coefficients displayed are related to these values, thereby allowing for the comparison between coefficients. The size of the coefficient factor gives an indication of how strongly the Y-variable (i.e., development, light exposure or UVB radiation) is correlated to each of the X-variables (i.e., metabolites) (BioPAT SIMCA user manual).

## Results

### Characterization of the Microclimates in the Canopy and Bunch Zones

**FIGURE 1:**
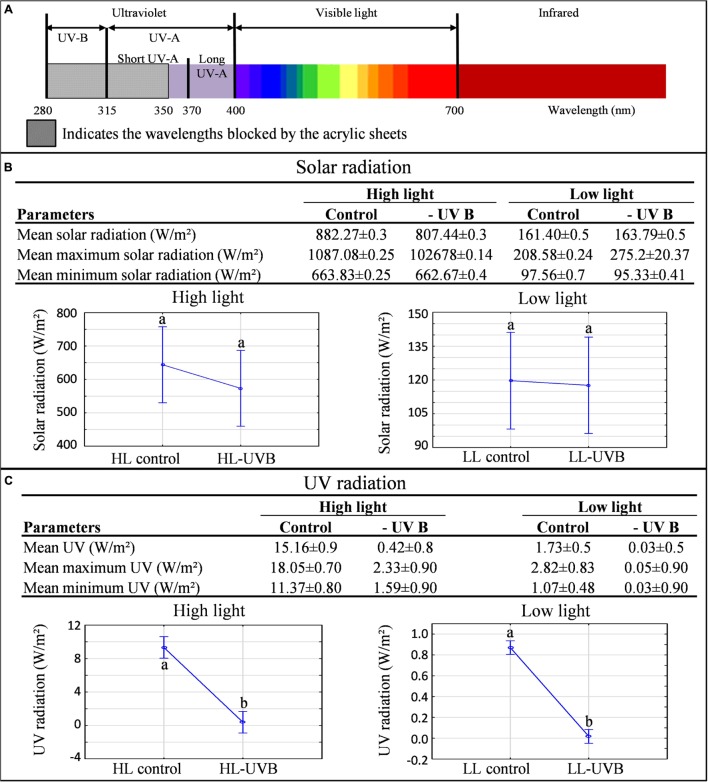
**A characterization of the light microclimates created by the four treatments in the 2014/2015 season **(A)**.** The electromagnetic spectrum showing the wavelengths blocked by the acrylic sheets used in the experiment. **(B)** The mean(±SD), mean maximum(±SD) and mean minimum(±SD) bunch solar radiation values **(B)** and bunch UV radiation values **(C)** calculated for each light environment over the sampling window (9h00-11h00) and their corresponding ANOVA plots; different letters indicate significant difference (*p* ≤ 0.05).

**FIGURE 2:**
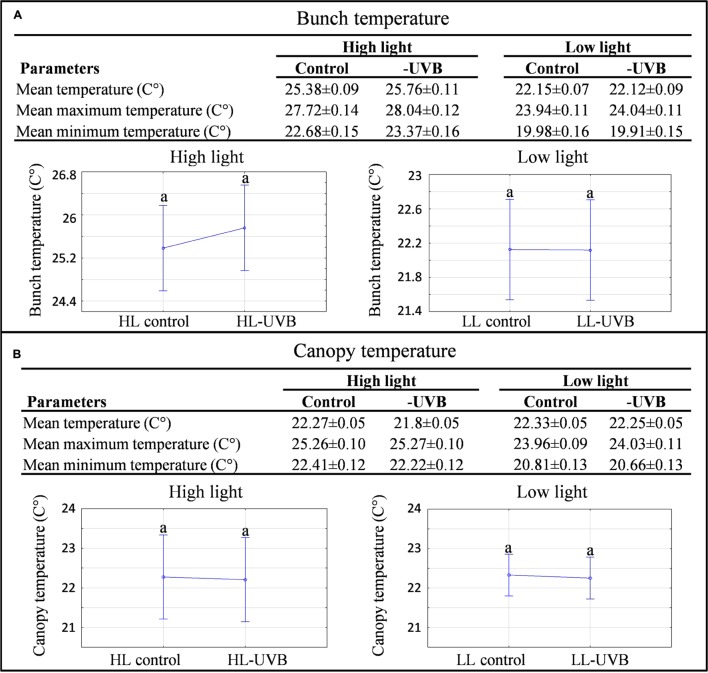
**A characterization of the temperature data collected in each microclimate in the 2014/2015 season.** The mean(±SD) mean maximum(±SD) and mean minimum(±SD) bunch **(A)** and canopy **(B)** temperatures measured on the sampling days during the sampling window (9h00 – 11h00) with the corresponding ANOVA plots for both high light and low light environments are shown; different letters indicate significant difference (*p* ≤ 0.05).

The characterization of the vineyard was performed according to the field-omics approach as explained in Alexandersson et al. (2014). Detailed monitoring was performed in the vineyard and the climatic data are summarized in **Table 1**, indicating that the targeted parameters for this study, namely solar radiation (including PAR) and UVB exposure significantly differed in the microclimates generated for this study (**Figure 1**; **Supplementary Figure S2**). The specifications of the acrylic sheets used stated that they would be able to block out 99% of UV light. This was confirmed by measuring the UV radiation behind and in front of the sheets. Further specification of these sheets can be seen in **Figure 1A**, indicating that the UV-excluding sheets would block UVB (280–315 nm) since it attenuated wavelengths between 280 and 350 nm. When evaluating the HL and LL environments separately, ANOVA plots furthermore showed that the HLcontrol and HL-UVB treatment (and similarly the LLcontrol and LL-UVB treatment) had similar solar radiation exposure levels, confirming that the UV-excluding sheets did not change the solar radiation further (**Figure 1B**). The data confirmed that the UV-excluding-sheets effectively attenuated UVB radiation reaching the bunch zone (**Figure 1C**). The leaf removal and increased exposure lead to differences in the bunch temperature between the HL and LL microclimates, but the UV-excluding sheets did not lead to additional differences in temperature within the HL (i.e., HLcontrol versus HL-UVB) or LL microclimates (**Figure 2**; **Supplementary Figure S3**). The canopy temperatures were similar between all four the experimental scenarios.

**Table 1:** A characterization of all the microclimatic climatic data collected in the 2014/2015 season on the sampling days during the sampling window (09h00 – 11h00).

		HLcontrol	HL-UVB	LLcontrol	LL-UVB
EL-31	Canopy temperature (°C)	24.4^a^	23.4^a^	24.3^a^	24.2^a^
	Bunch temperature (°C)	25.2^a^	25.4^a^	24.1^b^	23.7^b^
	Solar radiation (W/m^2^)	643.8^a^	707.8^a^	86.0^b^	86.8^b^
	UV (W/m^2^)	6.5^a^	0.4^b^	0.7^c^	0.0^d^
	Humidity (%)	57.1^a^	48.5^b^	59.1^c^	60.7^c^
EL-35	Canopy temperature (°C)	23.4^a^	22.8^a^	23.6^a^	23.6^a^
	Bunch temperature (°C)	29.9^a^	29.8^a^	23.7^b^	23.0^b^
	Solar radiation (W/m^2^)	998.7^a^	855.1^a^	201.3^b^	198.0^b^
	UV (W/m^2^)	8.6^a^	0.6^b^	0.8^c^	0.0^d^
	Humidity (%)	48.8^a^	39.0^b^	49.4^a^	53.0^c^
EL-38	Canopy temperature (°C)	19.0^a^	18.5^a^	19.0^a^	19.0^a^
	Bunch temperature (°C)	21.1^a^	22.0^a^	18.6^b^	18.7^b^
	Solar radiation (W/m^2^)	168.2^a^	156.7^a^	71.7^b^	68^b^
	UV (W/m^2^)	12.8^a^	0.2^b^	1.0^c^	0.0^d^
	Humidity (%)	71.9^a^	62.0^b^	68.6^c^	70.0^a^


*The table shows the mean values calculated over the sampling window per stage for each climatic variable and each individual light environment. Different superscripted letters indicate significant differences between variables: *p*-value < 0.001 ^a^; 0.001 < *p*-value < 0.01^b^; 0.01 < *p*-value < 0.05^c^ and insignificant ^d^.*

### Developmental and Treatment Impacts on Berry Metabolites

The ripening parameters showed typical developmental curves for grapevine berries (**Supplementary Figure S4**) with some variation in the total acids between seasons and samples at the earlier time-points.

When analyzing the berry metabolites from the first season of study using a repeated measures ANOVA (**Supplementary Table S3**), developmental stage had the strongest effect on chlorophyll, carotenoid and xanthophyll pool sizes, and the latter two pools were also significantly affected by both the exposure of the berries, as well as UVB attenuation. These results prompted a more in-depth analysis in a subsequent season on the photosynthetically related pigments, as well as volatile compounds in reaction to UVB attenuation. All the metabolite data measured over the two seasons in the green, véraison and ripe berries sampled from the four microclimates (HLcontrol, HL-UVB, LLcontrol, and LL-UVB) are provided in **Supplementary Table S4**.

Orthogonal partial least squares – discriminant analysis plots using developmental stage (**Supplementary Figure S5A**) or light exposure (**Supplementary Figure S5B**) as Y- variables, and the corresponding coefficient plots of compounds that contributed most to the models, highlighted metabolites that responded to the two factors. Separation in the samples was observed according to developmental stage with both primary and secondary metabolites contributing, in varying degrees, to the observed separation. Similarly, variation in light exposure also resulted in a clear separation between samples, confirming the influence of a HL and LL environment on berry metabolism (**Supplementary Figure S5B**). The metabolites mainly responsible for the separation, the xanthophylls, were similar to those previously reported by Young et al. (2016).

**FIGURE 3:**
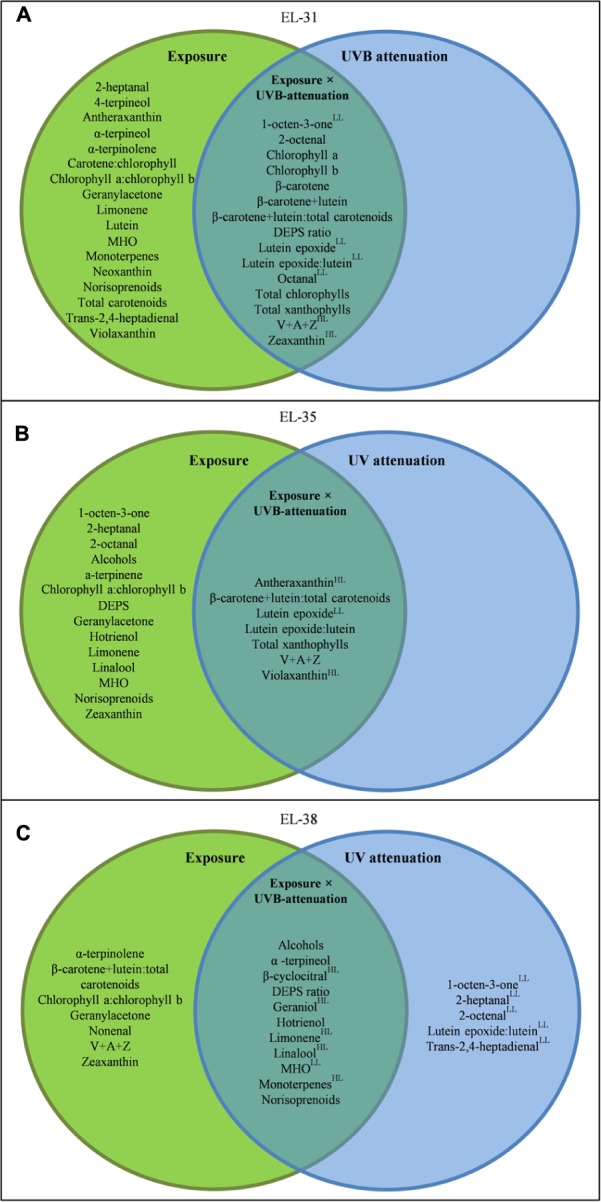
**A Venn diagram showing the compounds which responded to light exposure (green circle), UVB attenuation (blue circle) and both (intercept) in the early **(A)**, véraison **(B)**, and late **(C)** developmental stages.** Compounds were selected based on significance in a repeated measures ANOVA and Fisher LSD *Post Hoc* tests (adjusted *p*-value, *q*-value ≤0.05). All metabolites presented have a *q*-value ≤0.05. Metabolites with a log_2_-fold-change of ≥0.5 are indicated by a “^HL^” for the high light- or “^LL^” for the low light microclimate.

To better elucidate the subtle effects of UVB attenuation, OPLS-DA plots were created for the early and late stages of development separately. It was clear that different metabolites contributed to the separation in the green (**Supplementary Figure S6A**) versus ripe berries (**Supplementary Figure S6B**). The corresponding coefficient plots of compounds that contributed most to the models, highlighted specific xanthophylls and volatile aroma compounds that responded to UVB radiation/attenuation. The results of the OPLS-DA were further statistically validated by multifactor analysis (repeated measures ANOVA) in order to rank the significance of each compound in response to the three main experimental factors (i.e., development, light exposure and UVB radiation) individually, and in combination (**Table 2**). To simplify and visualize the data according to the main focus of the study (“What is the impact of UVB on berry metabolites and how is it different from exposure?”); compounds that responded to the variation in light exposure and/or UVB-attenuation were used to create Venn diagrams per developmental stage (**Figure 3**). Fisher LSD *Post Hoc* tests were used to identify statistically significant changes. Interestingly, in the pre-ripening stages, all compounds that responded to exposure, also responded to UVB attenuation. These compounds therefore differed in amplitude, and not in presence or absence. In the ripening stage, however, compounds were identified that responded only to UVB attenuation.

**Table 2:** An analysis of the photosynthetic pigments and volatile aroma compounds (2014/2015 season).

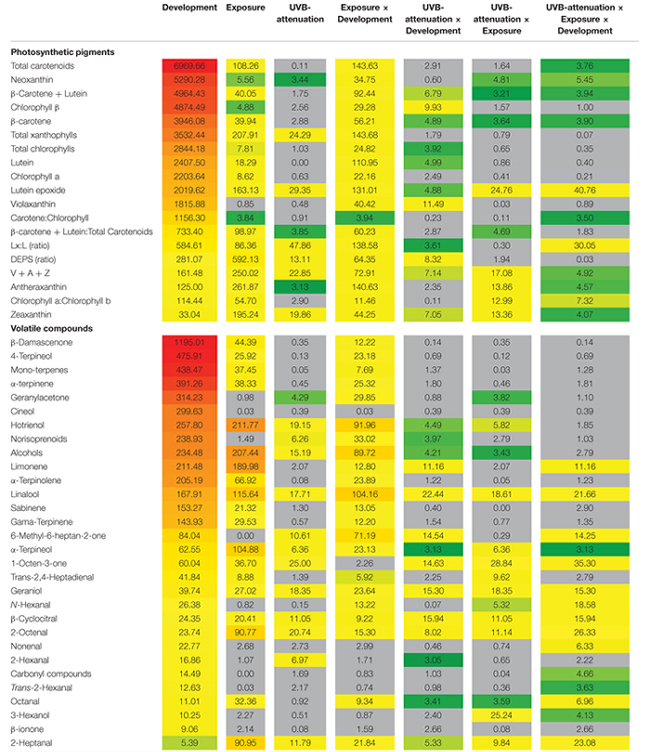

*The repeated measures ANOVA results for the listed parameters and individual compounds are reported as *F*-values. Values are scaled from highest (i.e., most significant) to lowest by color. Green indicates low *F-*values, while red indicates high *F*-values values. All insignificant values (*F* ≤ 3) are colored in gray. Maximum 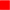; 50% 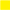; minimum 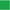; insignificant 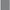.*

### Specific Xanthophylls Responded to UVB Attenuation in Predominantly the Green Photosynthetically Active Berry Stages

**FIGURE 4:**
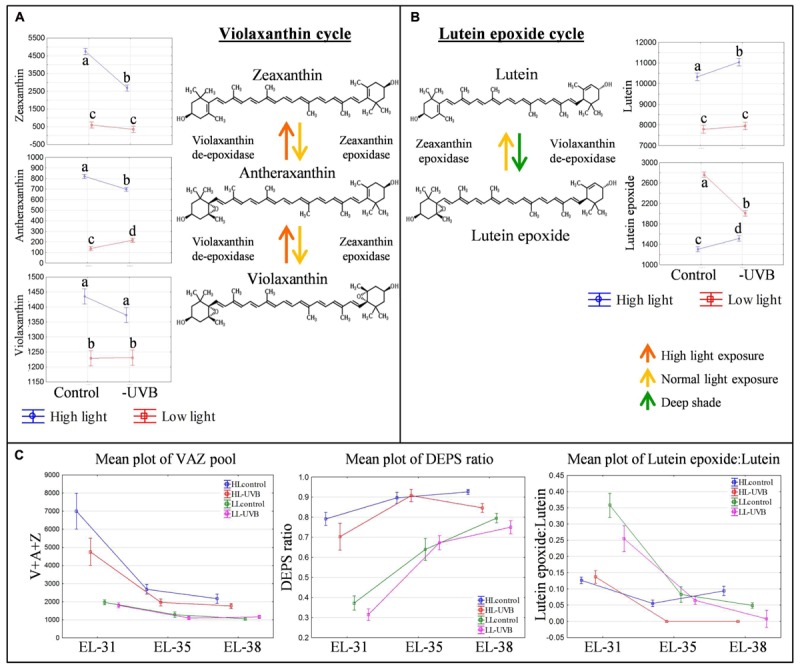
**The violaxanthin **(A)** and lutein epoxide **(B)** cycles with the ANOVA results for their associated xanthophylls in the green developmental stage (EL-31).** Different letters indicate significant difference (*p* ≤ 0.05). The mean plots of the associated xanthophyll pool (V + A + Z), DEPS ratio and lutein epoxide:lutein ratio for both high- and low light environments over all developmental stages **(C)**.

During the early stages of development, the xanthophylls zeaxanthin and lutein epoxide were identified as being the most responsive to UVB attenuation. Interestingly, the responses to UVB attenuation differed between the HL and LL environments. The attenuation of UVB in the HL environment resulted in a statistically significant decrease in zeaxanthin (**Figure 4**). This in turn resulted in a smaller xanthophyll pool size (violaxanthin, antheraxanthin, zeaxanthin) and a consequent lowered de-epoxidation state (DEPS ratio) in those samples (**Figure 4**). Although this was particularly obvious at the green berry stage, the lower xanthophyll pool, and consequent lower DEPS ratio, was consistently seen throughout berry development in the HL-UVB microclimate, but decreasing with developmental stage progression. Furthermore, the attenuation of UVB in the LL environment also resulted in a decreased V + A + Z pool and a lowered DEPS ratio in the green stage (**Figure 4**), although the effect was less pronounced compared to HL.

A significant difference in the levels of lutein epoxide between the LLcontrol and LL-UVB contrasts was also confirmed, clearly showing that UVB exposure in LL conditions is involved in the metabolism of lutein epoxide. Since lutein levels did not change, the Lx:L ratio was consequently significantly affected in the green developmental stage and to a lesser degree at the harvest stage (**Figure 4**).

### In the Ripe Berry Stages Specific Volatiles Responded to UVB Attenuation

**FIGURE 5:**
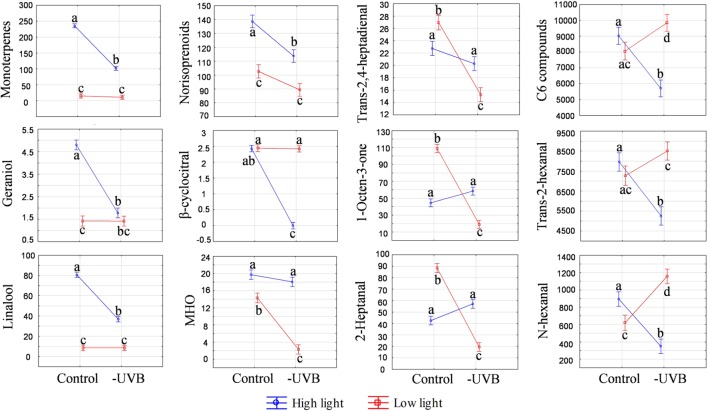
**Analysis of variance (ANOVA) plots for selected volatile compounds, including monoterpenes, norisoprenoids and C6 compounds measured at the late developmental stage (EL-38).** The results for both high light and low light environments are represented. Different letters indicate significant difference (*p* ≤ 0.05).

UVB attenuation was shown to affect specific volatile compounds in the ripe developmental stage (EL-38). These included monoterpenes, carotenoid-derived norisoprenoids and certain C_6_ compounds. In the HL environment, certain monoterpenes and norisoprenoids were decreased by UVB attenuation, leading to larger monoterpene and norisoprenoid pools in the HL control samples (**Figure 5**) and confirming that UVB exposure stimulates volatile organic compounds (VOCs) in exposed berries. Under LL conditions, however, both the monoterpene and norisporenoids pools were decreased relative to the HL microclimate and UVB attenuation resulted in no further statistically significant differences between the LLcontrol and LL-UVB microclimates.

Interestingly, under LL conditions, different VOC profiles as well as contents of individual volatile compounds were observed when comparing the LLcontrol with the UVB attenuated microclimate (LL-UVB) in ripe berry samples. Certain straight chain aldehydes and ketones (e.g., 1-octen-3-one, 2-heptanal and trans-2,4-heptadienal), decreased with UVB attenuation. Conversely, a significantly higher concentration of C_6_ compounds, including trans-2-hexenal and *N*-hexanal were observed when UVB was attenuated in the LL environment. This is the opposite of the scenario in HL, where the HLcontrol had more total C_6_ compounds than the HL-UVB (**Figure 5**).

**FIGURE 6:**
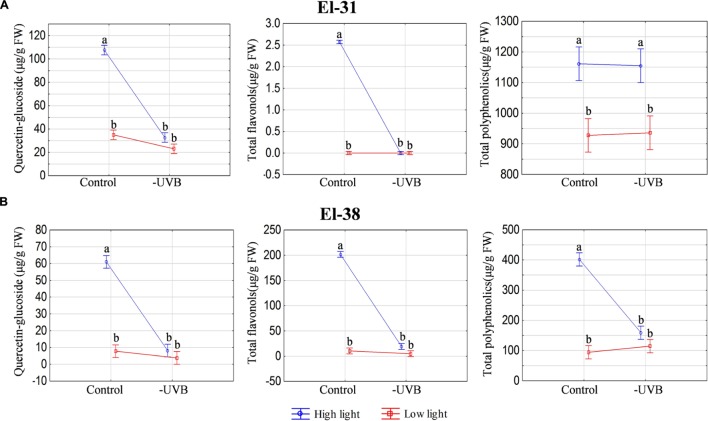
**The ANOVA plots for quercetin-glucoside, total flavonols and total polyphenolics measured at the early (EL-31) **(A)** and late developmental stage (EL-38) **(B)**.** The results for both high light and low light environments are represented. Quercetin-glucoside is expressed in μg/g fresh weight. The pooled compounds are expressed relative to quercetin-glucoside (μg/g fresh weight). Different letters indicate significant differences (*p* ≤ 0.05).

Furthermore, to control for well-known metabolite responses to UV, samples were also analyzed for polyphenols. As expected, total polyphenolics, and specifically the flavonol quercetin-glucoside, was significantly reduced with UVB attenuation in the HL microclimate, most notably in the early developmental stages (**Figure 6A**), although this pattern followed through to harvest (**Figure 6B**). No statistical significances were seen in the LL microclimate (LLcontrol versus LL-UVB) in either the early or late developmental stages.

## Discussion

A number of studies have shown that increased exposure (including UV) of grape berries, leads to the increased accumulation of polyphenolic compounds (Tardaguila et al., 2010; Diago et al., 2012; Song et al., 2015), as well as changes to varietal aroma compounds (Bureau et al., 2000; Zhang et al., 2014; Song et al., 2015). The increase in phenolic compounds, including anthocyanins, proanthocyanidins and flavonols, have been attributed to the increased expression of a number genes involved in their biosynthesis as a way to adapt to HL environments (Matus et al., 2009; Azuma et al., 2012). Carbonell-Bejerano et al. (2014) demonstrated that UV radiation upregulated a number of genes encoding transcription factors (e.g., MYBs and bHLH) that in turn activated flavonol biosynthetic genes [putative lyases, chalcone synthases, flavonol synthases (FLS) and flavonol glycosyltransferases] in grape berries. FLS is a dedicated enzyme involved in flavonol biosynthesis (e.g., quercetin) and its transcriptional response to light has been demonstrated in Shiraz (Downey et al., 2004).

In this study the characterization of the microclimates confirmed exposure and UVB attenuation as the main treatment effect in both the HL and LL environments. Marked increases in quercetin-glucoside contributed to a higher content of total polyphenolics in ripe berries in the HLcontrol (compared to HL-UVB), but not in the LL microclimate (**Figure 6**). The study illustrates that grapevine berries utilize polyphenolics as well as photosynthesis-related pigments in acclimation responses. These responses are differentially affected by UVB attenuation under HL and LL conditions in the different berry developmental stages. Since the carotenoid pigments are substrates for the formation of volatile aroma compounds (norisoprenoids) as ripening progresses, these volatile berry metabolites were also followed.

### Grapevine Berries Displayed Metabolic Plasticity in their Response to Attenuated UVB and the Response Was Influenced by the Developmental Stage of the Berries

In the green berry stage (EL-31) the xanthophylls reacted to the variations in UVB. This modulation of xanthophylls in the photosynthetically active green berries indicated that within the field setting, acclimation to light stress occurred in the early developmental stages. The data showed that the violaxanthin- and the lutein epoxide cycles were functional in the photosynthetically active berries in the HL and LL microclimates. The amplitudes of the cycles were, however, responsive to solar radiation and UVB. Although these cycles appear to be functional in the photosynthetically active green berries, and are typically regarded as photo-protective measures, the major carotenoids and chlorophylls were not significantly affected (log_2_-fold change ≤0.5) in either microclimate (HL or LL). This implies that the stress perceived by the photosynthetically active berries in the early developmental stages was mitigated by, for e.g., photoprotective mechanisms (e.g., non-photochemical quenching via the violaxanthin cycle) and photosynthesis was apparently unaffected (i.e., no evidence of photoinhibition and/or photodamage based on the core photosynthetic pigments). In the absence of UVB radiation, the berries required less zeaxanthin in HL microclimates, and conversely, less lutein epoxide in LL microclimates, to cope with the perceived stress and maintain active photosynthesis. The attenuation of UVB, however, potentially renders the plants more susceptible to damage as they are less acclimated than those plants exposed to UVB, especially in the LL microclimate. From numerous studies on photosynthetic organisms/tissues, it is known that the xanthophylls respond to light by way of the violaxanthin and/or lutein epoxide cycles (Demmig-Adams and Adams, 1996; García-Plazaola et al., 2007).

The photosynthetic efficiency of plants depends on their ability to adapt to natural daily variations in photon flux density. It is important that the photosynthetic plant tissues are able to absorb solar light and transfer the resulting energy to the relevant reaction centers under any light conditions. The light environment within a canopy is not fixed, but fluctuates in occurrence with the creation of gaps in the canopy or climatic changes (e.g., cloud cover). The alterations in the light environment may be transitory (e.g., sunflecks), or more permanent (e.g., leaf removal). In response to the variations in light exposure, plants have developed several morphological, physiological and biochemical mechanisms to optimize the light harvesting process as well as to protect the photosystems and maintain optimal functioning (Walters and Horton, 1994; Demmig-Adams and Adams, 2006; Johnson et al., 2007; García-Plazaola et al., 2007; Vogelmann and Gorton, 2014). It is evident that berries have maintained this photoprotective ability and respond to stress in the same way as photosynthetically active leaves.

In the HL microclimate, UVB-exposure lead to increased production of berry volatiles (predominantly monoterpenes including geraniol, linalool and limonene with a log_2_-fold change >1) in the later stages of berry development (from véraison onward). Similar results were seen in Malbec berries in that increased UVB exposure resulted in an increase in monoterpene emissions at the pre-harvest developmental stage. These results were interpreted to suggest that monoterpenes were involved in protection from UVB radiation (Gil et al., 2013). The antioxidant potential of terpenes (isoprene, monoterpenes, sesquiterpenes and tetraterpenes such as carotenoids) is well documented (Loreto and Velikova, 2001; Loreto et al., 2004) and it is possible that this is one of their biological functions in older (sink) tissues (such as ripe berries and/or senescing tissues).

A similar result was seen in the norisoprenoids in the HL environment with the most responsive of them being β-cyclocitral. In a LL environment, MHO was seen to react in a similar way in that it was significantly reduced by the attenuation of UVB. Norisoprenoids are formed via the degradation of carotenoids and the higher carotenoid content in HLcontrol berries may have directly resulted in the increased levels of norisoprenoids. Additionally, the derivatives of certain carotenoids are known to perform signaling functions in plants. Ramel et al. (2012) reported the rapid accumulation of β-cyclocitral upon exposure of *Arabidopsis* plants and the consequent reprogramming of gene expression to increase the capacity for photooxidative stress tolerance. The results of that study indicated that β-cyclocitral may serve as a signaling compound in plants which leads to the activation of oxidative stress defense mechanisms. Volatile carotenoid derivatives may therefore serve as sensing and signaling compounds when plants are subjected to stress as a way to mitigate potential damage. VOCs have been shown to increase in response to certain abiotic stresses (Possell and Loreto, 2013). It is speculated that volatile terpenes (e.g., monoterpenes) play important roles in the protection of plants from environmental stress (Loreto and Schnitzler, 2010; Carvalho et al., 2015). Although the exact mechanism is still unclear, the consistency of these links with stress warrants further investigation.

The higher C_6_-compounds levels (e.g., *n*-hexanal, *trans*-2-hexanal) in the HLcontrol berries (versus the HL-UVB berries), indicates a role for UVB in the regulation and/or metabolism of these compounds. Leaf removal is typically used in viticulture as a canopy management strategy to reduce the “green/vegetal” character of especially red cultivars (e.g., Cabernet Sauvignon). This green character is typically associated with pyrazines (predominantly methoxypyrazines), but can also be attributed to certain C_6_-compounds (e.g., hexanal) and some monoterpenes (e.g., eucalyptol) (Allen et al., 1991; Fariña et al., 2005; Lund et al., 2009). C_6_-compounds are produced via the lipoxygenase-hydroperoxide lyase (LOX-HPL) pathways and are developmentally regulated and known to be released during maceration or damage. Here we show that the UVB component of light contributes to the release of C_6_ compounds implicating UV in the regulation the LOX-HPL pathway and consequently the metabolism of polyunsaturated fatty acids (PUFAs). Interestingly, in the LL environment in the later developmental stages, the LLcontrol berries had significantly lower levels of the C_6_-compounds relative to the LL-UVB.

Attenuation of UVB in the LL environment decreased the levels of a number of straight chain aldehydes (e.g., 2-heptanal and *trans*-2,4-heptadienal) and a ketone (1-octen-3-one). These compounds therefore reacted similarly to the C_6_ compounds in the HL environment, and again implicating UVB in the metabolism of PUFAs. It is clear that the level of light exposure will determine which substrates are metabolized and/or which compounds are formed in berries, displaying considerable plasticity in these responses.

### Control Processes Over Non-photochemical Quenching, Photodamage and Photorepair Are Activated as Part of the Acclimation Responses and UVB Plays a Key Role

The increase in epoxidation state of the xanthophylls (as determined by the DEPS ratio) in the HL berries is due to higher zeaxanthin levels (versus violaxanthin) in the xanthophyll pool, and is indicative of a photosynthetic system that is utilizing non-photochemical quenching via zeaxanthin in the violaxanthin cycle. The response in the absence of UV (HL-UVB berries) is less than the HLcontrol, even though the incident PAR and bunch temperature are not significantly different. UVB exposure affects the amplitude of the violaxanthin cycle response (DEPS ratio due to different zeaxanthin levels). UVB radiation is known to affect the translation of psbA (D1 protein) in the photodamage/photorepair cycle, it is likely that in the absence of UVB (as in the HL-UVB), the photosystems recover quicker (via photorepair of photodamage) than in the presence of UVB radiation (as in the HLcontrol), and/or that the actual level of saturating conditions for photosynthesis are lower in the presence of UVB radiation and HL. These results provide a hypothesis for subsequent studies on UV effects on fruit physiology and metabolism and are supported by literature from a number of fruits (Arakawa et al., 1985; Ubi et al., 2006; Calvenzani et al., 2010; Huyskens-Keil et al., 2012).

Additionally the lutein epoxide cycle is lower in the UVB attenuated LL treatments (LL-UVB). Lutein epoxide is formed in shade (deep/long term shade) and functions to protect the photosynthetic apparatus from sudden localized HL exposures (e.g., sunflecks). Although the PAR in the LLcontrol and the LL-UVB were similar (low but differing only in the incident UVB), the lutein epoxide cycle is less active in the absence of UVB (LL-UVB). It appears as if it is the UVB component of solar radiation that is required for the formation of lutein epoxide (and by extension the functioning of the lutein epoxide cycle in LL microclimate). It is evident that both cycles are required and simultaneously functional in photosynthetically active berries (albeit to varying degrees) to potentially cope with the continuously varying light conditions in the microclimate: zeaxanthin in HL and lutein epoxide in LL, with UVB affecting the absolute amounts present in photosynthetically active berries.

These responses to varying light conditions are well known and well described in photosynthetic research on photosynthetic organs (predominantly leaves); but the reports for the response of fruit to UVB exposure appears to be limited to the formation of metabolites with antioxidant or “sunscreen” activity (polyphenolics, anthocyanins, flavonols, etc.). Increased exposure of the grape berries has been shown to result in the increase of polyphenolics and certain aromatic compounds in the berry tissues (Bureau et al., 2000; Tardaguila et al., 2010; Diago et al., 2012; Gil et al., 2013; Zhang et al., 2014; Song et al., 2015). It is tempting to speculate that the formation of these latter compounds represent molecular fingerprints of long term acclimation responses of early stage (i.e., photosynthetically active) fruits attempts at protecting photosynthesis distally (by reflecting incident radiation in predominantly the exposed skins and/or via general antioxidants to mitigate the damage of reactive oxygen species). The carotenoids (specifically the xanthophylls: zeaxanthin, antheraxanthin and lutein epoxide), however, are intrinsically linked to photosynthesis and are therefore probably the more direct/local response to saturating light conditions on the photosynthetic process (as on-site antioxidants or by direct non-photochemical quenching of reactive oxygen species). It could be that it is the failure of carotenoids and other lipophilic antioxidants present in the photosynthetic membranes (of green berries), to mitigate stress that trigger the long(er) term responses involving acclimation and other photomorphogenic responses to deal with the consequence of continued photodamage (e.g., structural changes to the skin composition and the accumulation of polyphenolics in the skin).

**FIGURE 7:**
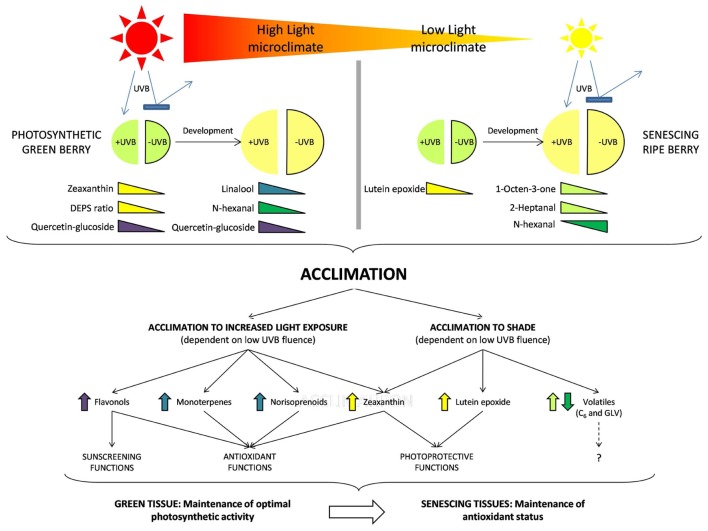
**A comprehensive model summarizing the results of the study.** In each light environment (HL and LL) both early and late developmental stages are represented as well as the attenuation of UVB. The colored triangles indicate those compounds which reacted to UVB attenuation in each case, indicating the presence of an acclimation response in the berries. Each of the compound groups perform a specific function in the berry tissue and contribute to the acclimation of the berry via various physiological processes. These processes differ depending on the tissue type and are therefore associated with the developmental stage of the berry.

The metabolic outcomes of these acclimation responses and the level of stress perceived in the different microclimates clearly impacts berry composition. It has been confirmed that in both leaves (Joshi et al., 2013; Juvany et al., 2013) and berries (Carbonell-Bejerano et al., 2014; Liu et al., 2015) young photosynthetically active tissues respond differently to increased exposure compared to older tissue (old, senescing leaves or ripe berries). **Figure 7** proposes an overview model of the respective responses and highlights the importance of the developmental stage (early or late) as well as the microclimate (HL or LL) on the metabolites that are differentially produced and proposed to play a role in the acclimation responses. The data presented supports the hypothesis that plants in shade are less acclimated and consequently more susceptible (on e.g., a clear day) than the exposed (HL) more acclimated counterparts (typically displaying higher flavonols, higher photo-protective xanthophylls, and/or antioxidant volatiles, depending on the developmental stage). In the absence of UVB, less acclimation has potentially occurred in the LL-UVB and the plants will be more susceptible (to e.g., sunflecks) than the more acclimated HL-UVB counterparts. Here we show that these general plant responses are active in grapevine berries with developmental stages displaying distinctive responses.

## Author Contributions

MV conceived and planned the study. CJ implemented and maintained the viticultural treatments and monitored the vineyard. CJ and PY carried out berry sampling. CJ did the climatic data processing and analysis, processing and analysis of the berry samples together with HE-B which performed the UPLC, HPLC, and GC-MS analysis. CJ performed the data integration and processing for the above compounds. CJ, HE-B, and PY performed data analysis. CJ, PY, and MV drafted the initial manuscript, all authors contributed to the final manuscript.

## Conflict of Interest Statement

The authors declare that the research was conducted in the absence of any commercial or financial relationships that could be construed as a potential conflict of interest.

## Supplementary Material

**FIGURE S1 | The experimental layout of the treatments within the plot **(A)** and the four light environments created by leaf removal and UVB attenuation **(B).****

**FIGURE S2 | The mean hourly seasonal (from berry set to harvest) solar radiation and UV radiation data (mean ± 95% confidence interval) for each light environment measured in the 2014/2015 experimental season.** The first hour is from 00h00 to 01h00.

**FIGURE S3 |The seasonal (2014/2015) bunch and canopy minimum, maximum, and mean (±SD) temperatures for all light environments and the corresponding kinetics showing the mean hourly bunch and canopy temperatures (mean ± 95% confidence interval) measured in the 2014/2015 experimental season.** The first hour is from 00h00 to 01h00.

**FIGURE S4 |** **(A)** The total sugars and total organic acid contents measured over berry development and the ripening parameters determined at harvest (2011/2012 season). **(B)** The ripening parameters measured for the last experimental season (2014/2015 season).

**FIGURE S5 | Orthogonal partial least squares – discriminant analysis (OPLS-DA) models generated for all metabolic data over both experimental seasons for developmental stage **(A)** and light exposure **(B)**.** Each OPLS-DA is accompanied by a co-efficient plot of compounds which contributed most to the respective models. These were chosen according to the individual variable importance plots (VIP’s) and included the top compounds with a VIP ≥ 0.5. Shapes of the sample icons denote the respective developmental stages: EL-31 (●), EL-35 () and EL-38 ().

**FIGURE S6 | Orthogonal partial least squares – discriminant analysis models generated for all metabolic data over both experimental seasons for the early **(A)** and late **(B)** developmental stages separately.** The attenuation of UVB was used as the y-factor in both models. Each OPLS-DA is accompanied by a co-efficient plot of compounds which contributed most to the respective models. These were chosen according to the individual variable importance plots (VIP’s) and included the top compounds with a value above 0.5. Shapes of the sample icons denote the respective exposure: High Light () and Low Light (●).

**TABLE S1 |Calibration curve of volatile organic compounds used in this study and analyzed by HS-SPME and GC single-quadrupole-MS.**

**TABLE S2 | Selected ions used for the integration of the peak area of the respective compounds of interest as well as their retention time on the Zebron column and quantifier molecules analyzed by HS-SPME and GC single-quadrupole-MS.**

**TABLE S3 | An analysis of the metabolic data from the first experimental season (2011/2012 season).** The repeated measures ANOVA results for the listed parameters and individual compounds are reported as *F*-values. Values are scaled from highest (most significant) to lowest by color. Green indicates low *F-*values (significant), while red indicates high *F*-values values (more significant). All insignificant values are highlighted in gray. Maximum ; 50% ; minimum ; insignificant .

**TABLE S4 | A table listing the measured contents of all the compounds ± SD for both experimental seasons.** The log_2_-fold changes and corresponding *p*-values between the HL control/HL-UVB and LL control)/LL-UVB contrasts are calculated and listed for each compound at each developmental stage. Blocks highlighted in red indicate significant difference (*p* ≤ 0.05).

## References

[B1] Alexandersson E. Jacobson D. Vivier M. A. Weckwerth W. Andreasson E. (2014). Field-omics—understanding large-scale molecular data from field crops. *Front. Plant Sci.* 5:286 10.3389/fpls.2014.00286 PMC4064663 24999347

[B2] Allen M. S. Lacey M. J. Harris R. L. Brown W. V. (1991). Contribution of methoxypyrazines to Sauvignon blanc wine aroma. *Am. J. Enol. Vitic.* 42 109–112.

[B3] Arakawa O. Hori Y. Ogata R. (1985). Relative effectiveness and interaction of ultraviolet-B, red and blue light in anthocyanin synthesis of apple fruit. *Physiol. Plant.* 64 323–327. 10.1111/j.1399-3054.1985.tb03347.x

[B4] Azuma A. Yakushiji H. Koshita Y. Kobayashi S. (2012). Flavonoid biosynthesis-related genes in grape skin are differentially regulated by temperature and light conditions. *Planta* 236 1067–1080. 10.1007/s00425-012-1650-x 22569920

[B5] Barros E. P. Moreira N. Pereira G. E. Leite S. G. F. Rezende C. M. de Pinho P. G. (2012). Development and validation of automatic HS-SPME with a gas chromatography-ion trap/mass spectrometry method for analysis of volatiles in wines. *Talanta* 101 177–186. 10.1016/j.talanta.2012.08.028 23158309

[B6] Blanke M. M. Lenz F. (1989). Fruit photosynthesis. *Plant Cell Environ.* 12 31–46. 10.1111/j.1365-3040.1989.tb01914.x

[B7] Bureau S. M. Razungles A. J. Baumes R. L. (2000). The aroma of muscat of Frontignan grapes: effect of the light environment of vine or bunch on volatiles and glycoconjugates. *J. Sci. Food Agric.* 80 2012–2020. 10.1002/1097-0010(200011)80:14<2012::AID-JSFA738>3.0.CO;2-X

[B8] Calvenzani V. Martinelli M. Lazzeri V. Giuntini D. Dall’Asta C. Galaverna G. (2010). Response of wild-type and high pigment-1 tomato fruit to UV-B depletion: flavonoid profiling and gene expression. *Planta* 231 755–765. 10.1007/s00425-009-1082-4 20033231

[B9] Carbonell-Bejerano P. Diago M.-P. Martínez-Abaigar J. Martínez-Zapater J. M. Tardáguila J. Núñez-Olivera E. (2014). Solar ultraviolet radiation is necessary to enhance grapevine fruit ripening transcriptional and phenolic responses. *BMC Plant Biol.* 14:183 10.1186/1471-2229-14-183 PMC4099137 25012688

[B10] Carvalho L. C. Coito J. L. Colaco S. Sangiogo M. Amancio S. (2015). Heat stress in grapevine: the pros and cons of acclimation. *Plant Cell Environ.* 38 777–789. 10.1111/pce.12445 25211707

[B11] Cramer G. R. Urano K. Delrot S. Pezzotti M. Shinozaki K. (2011). Effects of abiotic stress on plants: a systems biology perspective. *BMC Plant Biol.* 11:163 10.1186/1471-2229-11-163 PMC3252258 22094046

[B12] Dal Santo S. Tornielli G. B. Zenoni S. Fasoli M. Farina L. Anesi A. (2013). The plasticity of the grapevine berry transcriptome. *Genome Biol.* 14:r54 10.1186/gb-2013-14-6-r54 PMC3706941 23759170

[B13] Demmig-Adams B. Adams W. W. (1996). The role of xanthophyll cycle carotenoids in the protection of photosynthesis. *Trends Plant Sci.* 1 21–26. 10.1016/S1360-1385(96)80019-7

[B14] Demmig-Adams B. Adams W. W. (2006). Photoprotection in an ecological context: the remarkable complexity of thermal energy dissipation. *New Phytol.* 172 11–21. 10.1111/j.1469-8137.2006.01835.x 16945085

[B15] Diago M. P. Ayestarán B. Guadalupe Z. Poni S. Tardáguila J. (2012). Impact of prebloom and fruit-Set basal leaf removal on the flavonol and anthocyanin composition of Tempranillo grapes. *Am. J. Enol. Vitic.* 63 367–376. 10.5344/ajev.2012.11116

[B16] Downey M. O. Harvey J. S. Robinson S. P. (2004). The effect of bunch shading on berry development and flavonoid accumulation in Shiraz grapes. *Aust. J. Grape Wine Res.* 10 55–73. 10.1111/j.1755-0238.2004.tb00008.x

[B17] Eichhorn K. W. Lorenz D. H. (1977). Phanologische entwicklungsstadien der rebe. *Nachr. Dtsch. Pflanzenschutzd.* 29 119–120.

[B18] Eyéghé-Bickong H. A. Alexandersson E. O. Gouws L. M. Young P. R. Vivier M. A. (2012). Optimisation of an HPLC method for the simultaneous quantification of the major sugars and organic acids in grapevine berries. *J. Chromatogr. B Analyt. Technol. Biomed. Life Sci.* 885–886, 43–49. 10.1016/j.jchromb.2011.12.011 22265666

[B19] Fariña L. Boido E. Carrau F. Versini G. Dellacassa E. (2005). Terpene compounds as possible precursors of 18-cineole in red grapes and wines. *J. Agric. Food Chem.* 53 1633–1636. 10.1021/jf040332d 15740051

[B20] Favory J. J. Stec A. Gruber H. Rizzini L. Oravecz A. Funk M. (2009). Interaction of COP1 and UVR8 regulates UV-B-induced photomorphogenesis and stress acclimation in *Arabidopsis*. *EMBO J.* 28 591–601. 10.1038/emboj.2009.4 19165148 PMC2657586

[B21] García-Plazaola J. I. Matsubara S. Osmond C. B. (2007). The lutein epoxide cycle in higher plants: its relationships to other xanthophyll cycles and possible functions. *Funct. Plant Biol.* 34 759–773. 10.1071/FP07095 32689404

[B22] Gil M. Bottini R. Berli F. Pontin M. Silva M. F. Piccoli P. (2013). Volatile organic compounds characterized from grapevine (*Vitis vinifera* L. *cv.* Malbec) berries increase at pre-harvest and in response to UV-B radiation. *Phytochemistry* 96 148–157. 10.1016/j.phytochem.2013.08.011 24075072

[B23] Gregan S. M. Wargent J. J. Liu L. Shinkle J. Hofmann R. Winefield C. (2012). Effects of solar ultraviolet radiation and canopy manipulation on the biochemical composition of Sauvignon Blanc grapes. *Aust. J. Grape Wine Res.* 18 227–238. 10.1111/j.1755-0238.2012.00192.x

[B24] Hideg E. Jansen M. A. K. Strid A. (2013). UV-B exposure, ROS, and stress: inseparable companions or loosely linked associates? *Trends Plant Sci*. 18 107–115. 10.1016/j.tplants.2012.09.003 23084465

[B25] Huyskens-Keil S. Eichholz I. Kroh L. W. Rohn S. (2012). UV-B induced changes of phenol composition and antioxidant activity in black currant fruit (*Ribes nigrum* L.). *J. Appl. Bot. Food Qual.* 81 140–144.

[B26] Johnson M. P. Havaux M. Triantaphylidès C. Ksas B. Pascal A. A. Robert B. (2007). Elevated zeaxanthin bound to oligomeric LHCII enhances the resistance of *Arabidopsis* to photooxidative stress by a lipid-protective, antioxidant mechanism. *J. Biol. Chem.* 282 22605–22618. 10.1074/jbc.M702831200 17553786

[B27] Joshi P. Nayak L. Misra A. N. Biswal B. (2013). “Response of mature, developing and senescing chloroplasts to environmental stress,” in *Plastid Development in Leaves during Growth and Senescence*, eds Biswal B. Krupinska K. Biswal U. C. (Amsterdam: Springer),641–668.

[B28] Juvany M. Müller M. Munné-Bosch S. (2013). Photo-oxidative stress in emerging and senescing leaves: a mirror image? *J. Exp. Bot.* 64 3087–3098. 10.1093/jxb/ert174 23825233

[B29] Kolb C. A. Kopecký J. Riederer M. Pfündel E. E. (2003). UV screening by phenolics in berries of grapevine (*Vitis vinifera*). *Funct. Plant Biol.* 30 1177–1186. 10.1071/FP03076 32689099

[B30] Lashbrooke J. G. Young P. R. Strever A. E. Stander C. Vivier M. A. (2010). The development of a method for the extraction of carotenoids and chlorophylls from grapevine leaves and berries for HPLC profiling. *Aust. J. Grape Wine Res.* 16 349–360. 10.1111/j.1755-0238.2010.00097.x

[B31] Li J. Yang L. Jin D. Nezames C. D. Terzaghi W. Deng X. W. (2013). UV-B-induced photomorphogenesis in *Arabidopsis*. *Protein Cell* 4 485–492. 10.1007/s13238-013-3036-7 23744340 PMC4875513

[B32] Liu L. Gregan S. Winefield C. Jordan B. (2015), From UVR8 to flavonol synthase: UV-B-induced gene expression in Sauvignon blanc grape berry. *Plant Cell Environ.* 38 905–919. 10.1111/pce.12349 24738597

[B33] Loreto F. Schnitzler J. P. (2010). Abiotic stresses and induced BVOCs. *Trends Plant Sci.* 15 154–166. 10.1016/j.tplants.2009.12.006 20133178

[B34] Loreto F. Velikova V. (2001). Isoprene produced by leaves protects the photosynthetic apparatus against ozone damage, quenches ozone products, and reduces lipid peroxidation of cellular membranes. *Plant Physiol.* 127 1781–1787. 10.1104/pp.010497 11743121 PMC133581

[B35] Loreto F. Pinelli P. Manes F. Kollist H. (2004). Impact of ozone on monoterpene emissions and evidence for an isoprene-like antioxidant action of monoterpenes emitted by *Quercus ilex* leaves. *Tree Physiol.* 24 361–367. 10.1093/treephys/24.4.361 14757575

[B36] Lund C. M. Thompson M. K. Benkwitz F. Wohler M. W. Triggs C. M. Gardner R. (2009). New Zealand Sauvignon blanc distinct flavor characteristics: sensory, chemical, and consumer aspects. *Am. J. Enol. Vitic.* 60 1–12.

[B37] Martínez-Lüscher J. Morales F. Delrot S. Sánchez-Díaz M. Gomés E. Aguirreolea J. (2013). Short- and long-term physiological responses of grapevine leaves to UV-B radiation. *Plant Sci.* 213 114–122. 10.1016/j.plantsci.2013.08.010 24157214

[B38] Matus J. T. Loyola R. Vega A. Peña-Neira A. Bordeu E. Arce-Johnson P. (2009). Post-veraison sunlight exposure induces MYB-mediated transcriptional regulation of anthocyanin and flavonol synthesis in berry skins of *Vitis vinifera*. *J. Exp. Bot.* 60 853–867. 10.1093/jxb/ern336 19129169 PMC2652055

[B39] Pontin M. A. Piccoli P. N. Francisco R. Bottini R. Martinez-Zapater J. M. Lijavetzky D. (2010). Transcriptome changes in grapevine (*Vitis vinifera* L.) cv. Malbec leaves induced by ultraviolet-B radiation. *BMC Plant Biol.* 10:224 10.1186/1471-2229-10-224 PMC3017828 20959019

[B40] Possell M. Loreto F. (2013). “The role of volatile organic compounds in plant resistance to abiotic stresses: responses and mechanisms,” in *Biology Controls and Models of Tree Volatile Organic Compound emissions*, eds Niinemets Ü. Monson R. K. (Amsterdam: Springer), 209–235.

[B41] Ramel F. Birtic S. Ginies C. Soubigou-Taconnat L. Triantaphylidès C. Havaux M. (2012). Carotenoid oxidation products are stress signals that mediate gene responses to singlet oxygen in plants. *Proc. Natl. Acad. Sci. U.S.A.* 109 5535–55 10.1073/pnas.1115982109 22431637 PMC3325660

[B42] Schultz H. R. Löhnertz O. Bettner W. Bálo B. Linsenmeier A. Jähnisch A. (1998). Is grape composition affected by current levels of UV-B radiation? *VITIS-J. Grapevine Res.* 37 191–192.

[B43] Song J. Smart R. Wang H. Dambergs B. Sparrow A. Qian M. C. (2015). Effect of grape bunch sunlight exposure and UV radiation on phenolics and volatile composition of *Vitis vinifera* L. cv. Pinot noir wine. *Food Chem.* 173 424–431. 10.1016/j.foodchem.2014.09.150 25466041

[B44] Steel C. C. Keller M. (2000). Influence of UV-B irradiation on the carotenoid content of *Vitis vinifera* tissues. *Biochem. Soc. Trans.* 28 883–885. 10.1042/bst0280883 11171244

[B45] Tardaguila J. de Toda F. M. Poni S. Diago M. P. (2010). Impact of early leaf removal on yield and fruit and wine composition of *Vitis vinifera* L. Graciano and Carignan. *Am. J. Enol. Vitic.* 61 372–381.

[B46] Thayer S. S. Björkman O. (1990). Leaf xanthophyll content and composition in sun and shade determined by HPLC. *Photosyn. Res.* 23 331–343. 10.1007/BF00034864 24419657

[B47] Tian B. Harrison R. Jaspers M. Morton J. (2015). Influence of ultraviolet exclusion and of powdery mildew infection on Sauvignon Blanc grape composition and on extraction of pathogenesis-related proteins into juice. *Aust. J. Grape Wine Res.* 21 417–424. 10.1111/ajgw.12135

[B48] Tilbrook K. Arongaus A. B. Binkert M. Heijde M. Yin R. Ulm R. (2013). The UVR8 UV-B photoreceptor: perception, signaling and response. *Arabidopsis Book* 11:e0164 10.1199/tab.0164 PMC3711356 23864838

[B49] Ubi B. E. Honda C. Bessho H. Kondo S. Wada M. Kobayashi S. (2006). Expression analysis of anthocyanin biosynthetic genes in apple skin: effect of UV-B and temperature. *Plant Sci.* 170 571–578. 10.1016/j.plantsci.2005.10.009

[B50] Vogelmann T. C. Gorton H. L. (2014). “Leaf: light capture in the photosynthetic organ,” in *The Structural Basis of Biological Energy Generation*, ed. Hohmann-Marriott M. F. (Amsterdam: Springer), 363–377.

[B51] Walters R. G. Horton P. (1994). Acclimation of *Arabidopsis thaliana* to the light environment: changes in composition of the photosynthetic apparatus. *Planta* 195 248–256. 10.1007/BF00199685 8547817

[B52] Young P. Eyeghe-Bickong H. A. du Plessis K. Alexandersson E. Jacobson D. A. Coetzee Z. A. (2016). Grapevine plasticity in response to an altered microclimate: sauvignon Blanc modulates specific metabolites in response to increased berry exposure. *Plant Physiol.* 170 1235–1254. 26628747 10.1104/pp.15.01775 PMC4775134

[B53] Zhang H. Fan P. Liu C. Wu B. Li S. Liang Z. (2014). Sunlight exclusion from Muscat grape alters volatile profiles during berry development. *Food Chem.* 164 242–250. 10.1016/j.foodchem.2014.05.012 24996330

