# Supplementary figures and images for: Field-Grown Grapevine Berries Use Carotenoids and the Associated Xanthophyll Cycles to Acclimate to UV Exposure Differentially in High and Low Light (Shade) Conditions
Source: Front Plant Sci. 2016 Jun 10;7:786. doi: 10.3389/fpls.2016.00786 (PMC4901986; doi:10.3389/fpls.2016.00786)

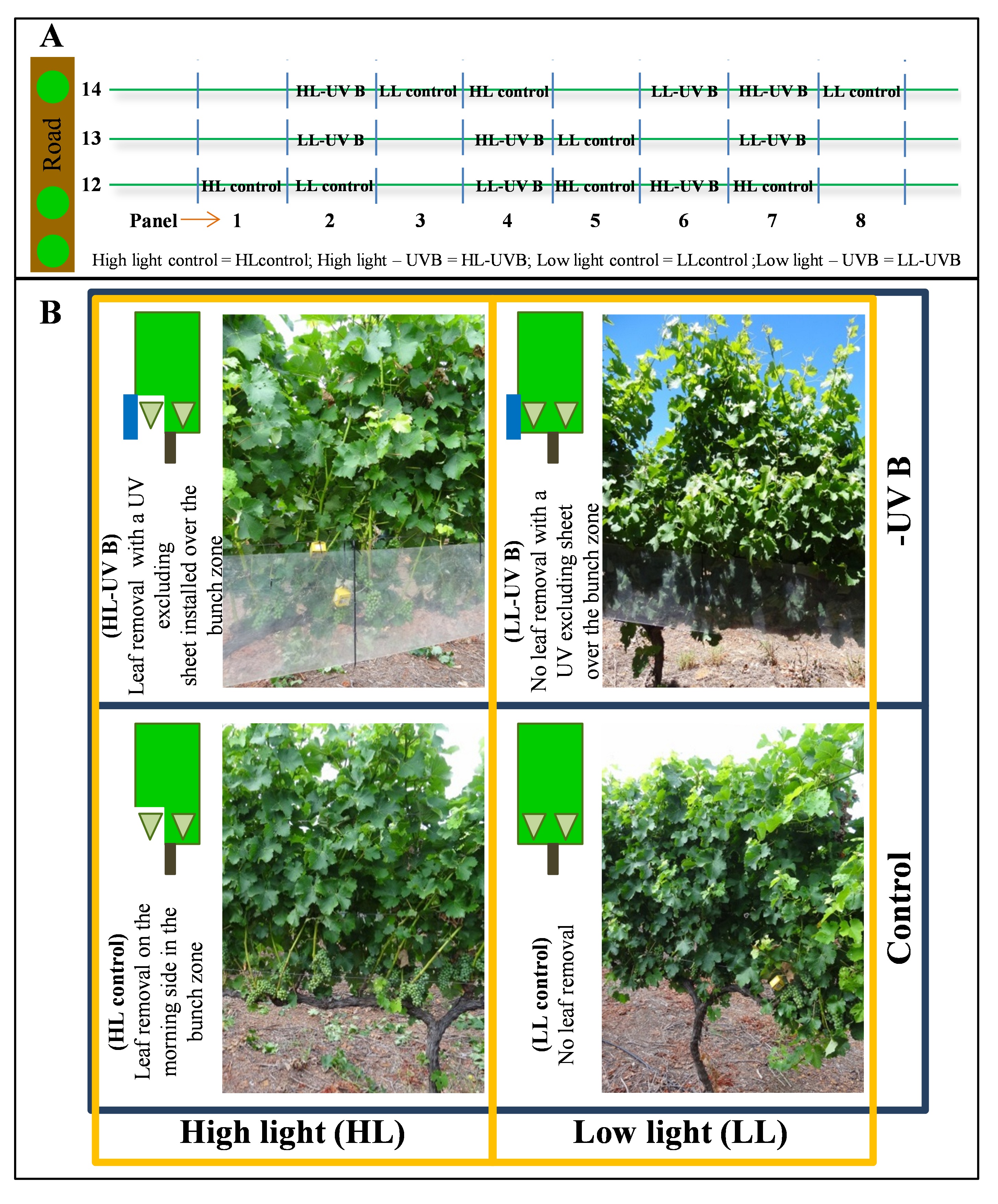

Supplement: Supplementary file 1 [file Image_1.JPEG]

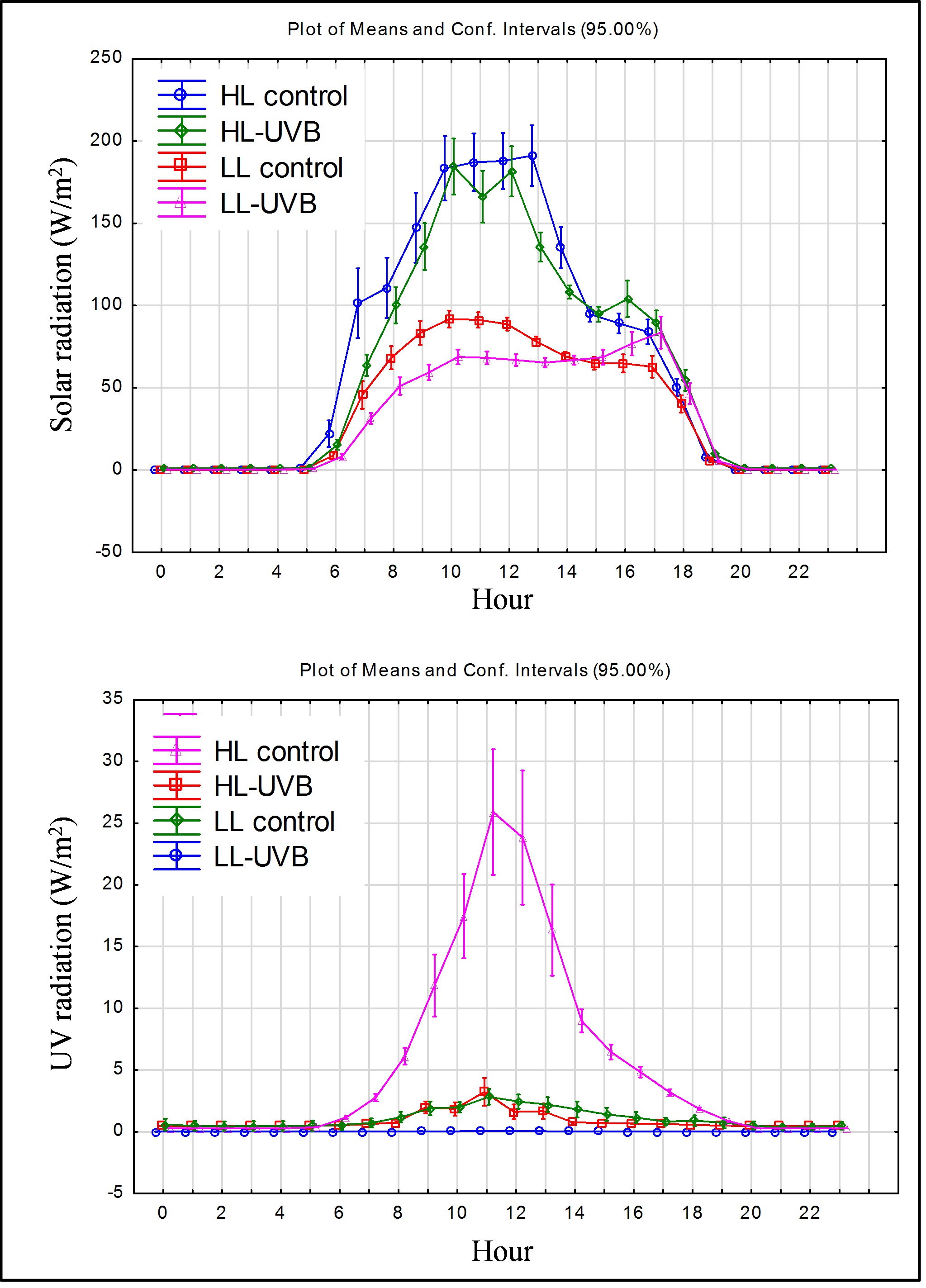

Supplement: Supplementary file 2 [file Image_2.JPEG]

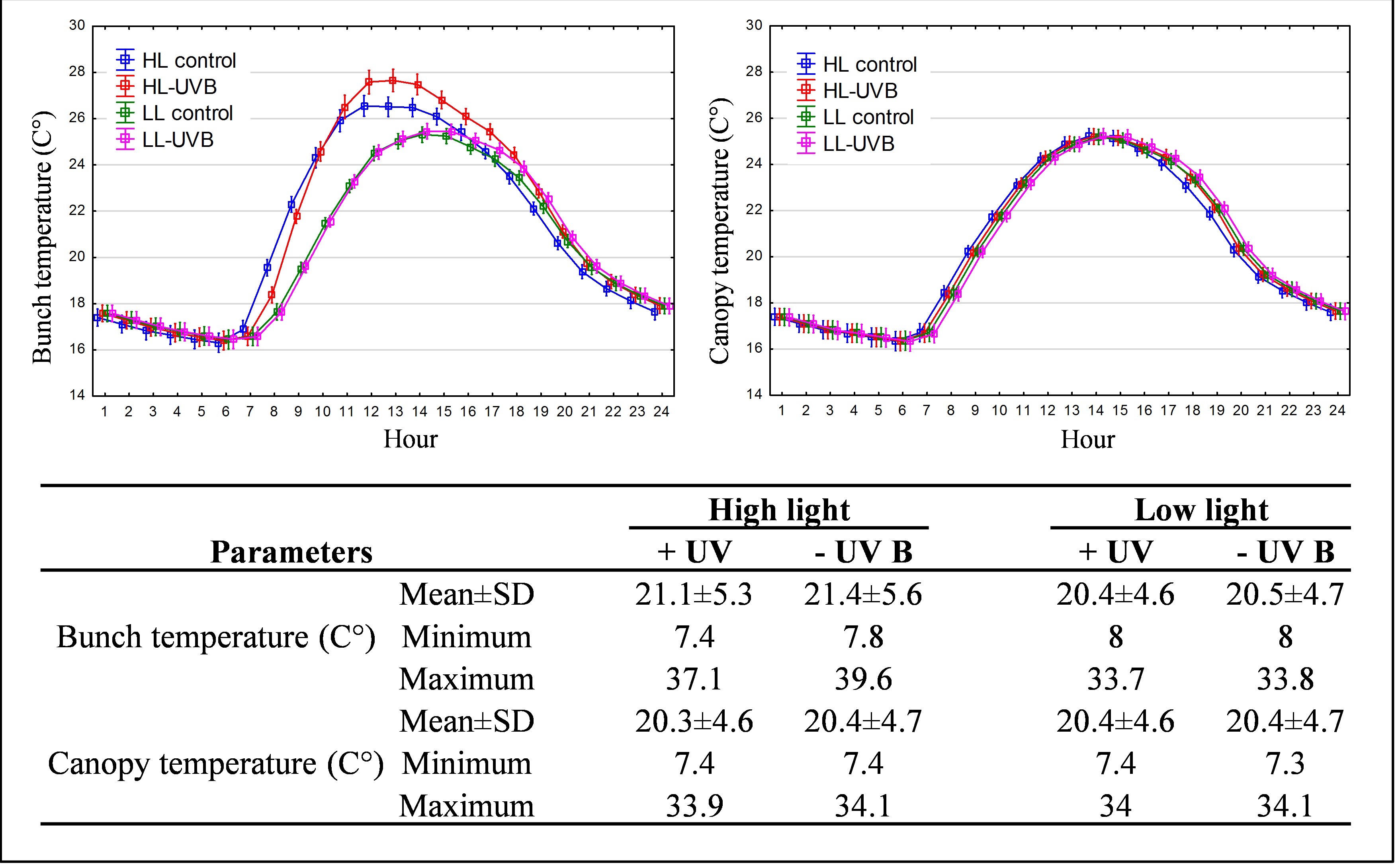

Supplement: Supplementary file 3 [file Image_3.JPEG]

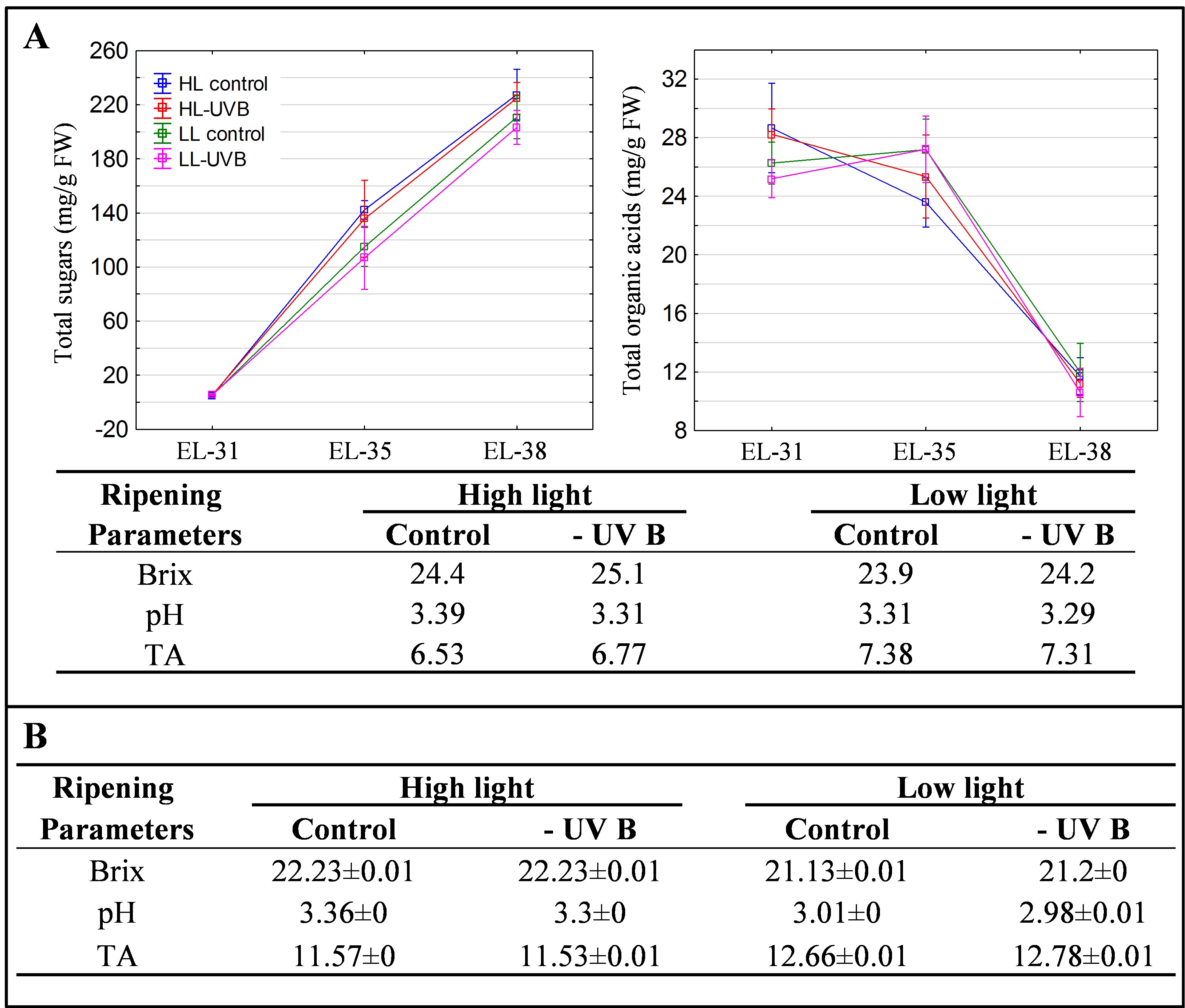

Supplement: Supplementary file 4 [file Image_4.JPEG]

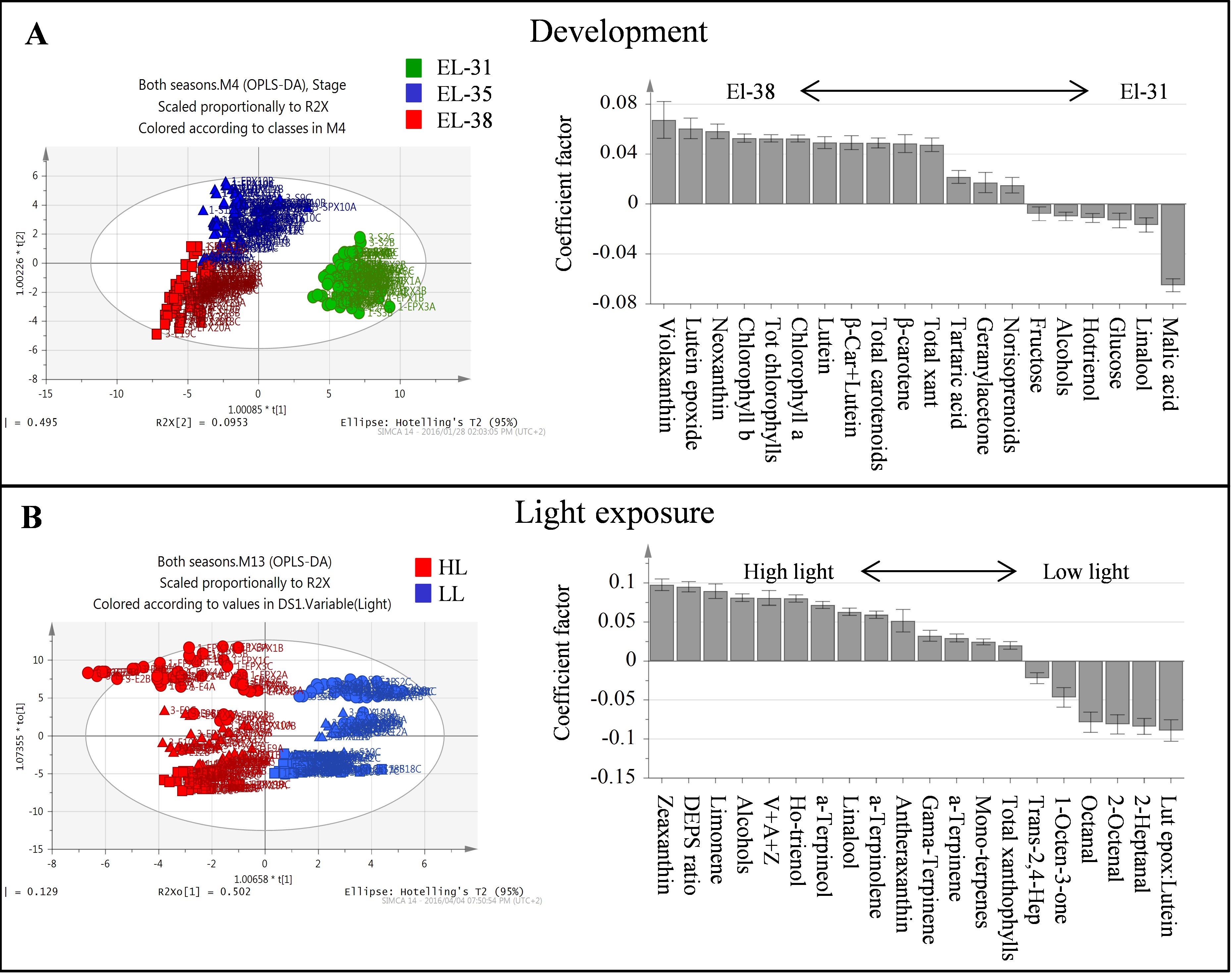

Supplement: Supplementary file 5 [file Image_5.JPEG]

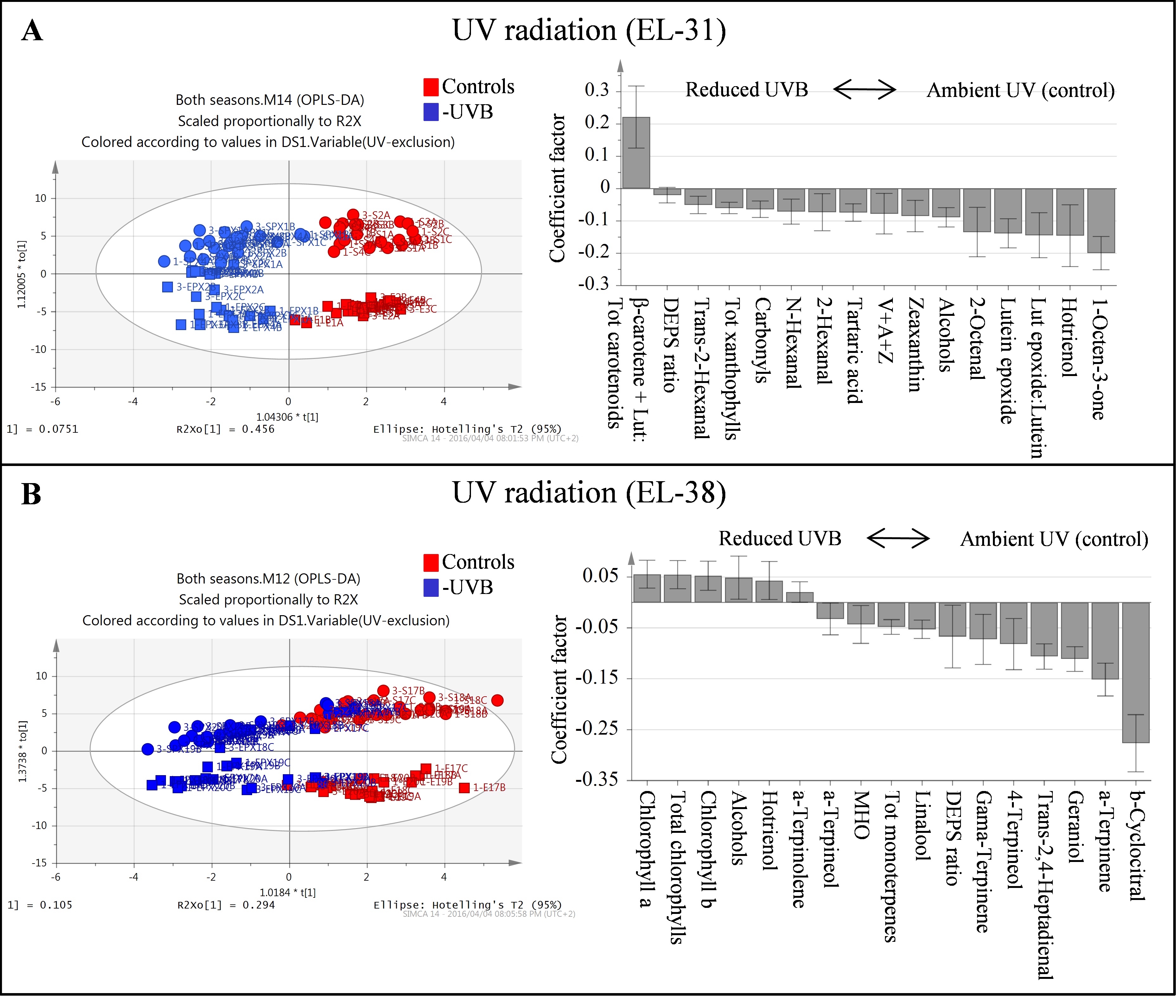

Supplement: Supplementary file 6 [file Image_6.JPEG]
