# Supplementary material for: Field-Grown Grapevine Berries Use Carotenoids and the Associated Xanthophyll Cycles to Acclimate to UV Exposure Differentially in High and Low Light (Shade) Conditions
Source: Front Plant Sci. 2016 Jun 10;7:786. doi: 10.3389/fpls.2016.00786 (PMC4901986; doi:10.3389/fpls.2016.00786)
Supplement: Supplementary file 7 [file Table_1.DOCX]

| **Compounds** | **Ion** | **Quantification range (µg/L)** | **y-intercept** | **Slope** | **LOD (µg/L)** | **LOQ (µg/L)** | **r^2^** |
| --- | --- | --- | --- | --- | --- | --- | --- |
| β-Damascone | 177 | 0-8.8 | -0.005 | 0.026 | 0.67 | 2.05 | 0.9965 |
| α-ionone | 177 | 0-8.9 | 0.000 | 0.001 | 0.51 | 1.55 | 0.9975 |
| β-ionone | 177 | 0-7.8 | -0.004 | 0.018 | 0.94 | 2.86 | 0.9916 |
| Limonene | 93 | 0.18.9 | 0.002 | 0.001 | 6.83 | 20.69 | 0.9225 |
| Linalool | 93 | 0-18.9 | 0.001 | 0.019 | 1.60 | 4.84 | 0.9960 |
| α-Terpineol | 93 | 0-4.7 | -0.001 | 0.044 | 0.25 | 0.76 | 0.9986 |
| Linalool-Oxide | 111 | 0-8.2 | 0.000 | 0.001 | 0.92 | 2.78 | 0.9927 |
| Trans-2-Hexanal | 83 | 0-95.7 | 0.005 | 0.002 | 20.02 | 60.68 | 0.9708 |
| 1-Hexanol | 55 | 0-10.5 | -0.001 | 0.015 | 0.37 | 1.11 | 0.9993 |
| 2-Heptenal | 55 | 0-40 | 0.005 | 0.010 | 4.83 | 14.63 | 0.9901 |
| MHO | 108 | 0-7.5 | -0.001 | 0.016 | 0.35 | 1.06 | 0.9987 |
| 2-Octanal | 55 | 0-24.6 | 0.000 | 0.020 | 1.05 | 3.17 | 0.9988 |
| 4-Terpineol | 71 | 0-10 | -0.008 | 0.076 | 0.31 | 0.93 | 0.9995 |
| Citronellol | 93 | 0-7 | -0.003 | 0.024 | 0.45 | 1.36 | 0.9976 |
| Nerol | 93 | 0-8.6 | 0.000 | 0.013 | 0.52 | 1.58 | 0.9979 |
| β-damascenone | 93 | 0-8 | -0.017 | 0.121 | 0.42 | 1.28 | 0.9984 |
| Geraniol | 93 | 0-6 | -0.003 | 0.051 | 0.29 | 0.89 | 0.9986 |
| Geranylactone | 93 | 0-34.4 | 0.005 | 0.023 | 0.89 | 2.99 | 0.9994 |
| Pseudo-ionone | 124 | 0-8.3 | 0.000 | 0.001 | 1.00 | 2.17 | 0.9958 |
