# Supplementary material for: Field-Grown Grapevine Berries Use Carotenoids and the Associated Xanthophyll Cycles to Acclimate to UV Exposure Differentially in High and Low Light (Shade) Conditions
Source: Front Plant Sci. 2016 Jun 10;7:786. doi: 10.3389/fpls.2016.00786 (PMC4901986; doi:10.3389/fpls.2016.00786)
Supplement: Supplementary file 8 [file Table_2.DOCX]

| **Compounds** | **RT (min)** | **Ion** | **Quantifier** |
| --- | --- | --- | --- |
| N-Hexanal | 4.76 | 82 | Trans-2-Hexanal |
| β-Myrcene | 4.94 | 93 | Limonene |
| α-Pinene | 5.78 | 93 | Limonene |
| α-terpinene | 5.96 | 93 | Limonene |
| Limonene | 6.26 | 93 | Limonene |
| Sabinene | 6.41 | 93 | Limonene |
| Cineol | 6.49 | 93 | Limonene |
| 2-Hexanal | 6.56 | 83 | Trans-2-Hexanal |
| Trans-2-Hexanal | 6.86 | 83 | Trans-2-Hexanal |
| Gama-Terpinene | 7.03 | 93 | Limonene |
| α-Terpinolene | 7.62 | 93 | Limonene |
| Octanal | 7.86 | 55 | 2-Octenal |
| 1-Octen-3-one | 8.05 | 55 | MHO |
| 2-Heptanal | 8.41 | 55 | 2-Heptanal |
| MHO | 8.58 | 108 | MHO |
| IS(Anisol) | 8.67 | 116 | - |
| 1-Hexanol | 8.77 | 55 | 1-Hexanol |
| 3-Hexanol | 9.20 | 55 | 1-Hexanol |
| Nonenal | 9.34 | 55 | 2-Octenal |
| Fenchone | 9.32 | 81 | Fenchone |
| 2,4-Hexadienal | 9.51 | 81 | Trans-2-Hexanal |
| 2-Octenal | 9.86 | 55 | 2-Octenal |
| Cis-Linolool oxide | 9.97 | 111 | Cis-Linolool oxide |
| Trans-Linalool oxide | 10.35 | 111 | Trans-Linalool oxide |
| Cis-2,4-Heptadienal | 10.38 | 81 | 2-Heptanal |
| Trans-2,4-Heptadienal | 10.77 | 81 | 2-Heptanal |
| 2-Nonenal | 11.23 | 93 | 2-Octenal |
| Linalool | 11.29 | 93 | Linalool |
| Junipene | 11.57 | 93 | Linalool |
| Trans-b-caryophyllene | 11.73 | 93 | Linalool |
| Trans-b-caryophyllene | 11.92 | 93 | Linalool |
| 4-Terpineol | 12.03 | 71 | 4-Terpineol |
| Hotrienol | 12.09 | 71 | 1-Hexanol |
| b-Cyclocitral | 12.29 | 123 | Linalool |
| 2-Decanal | 12.50 | 93 | 2-Octenal |
| α-Humulene | 12.81 | 93 | Linalool |
| α-Humulene | 12.87 | 93 | Linalool |
| a-Terpineol | 13.14 | 93 | a-Terpineol |
| Trans-trans-nona-2,4-dienal | 13.31 | 81 | 2-Octenal |
| α-Farnesene | 13.54 | 93 | a-Terpineol |
| EE-α-Farnesene | 13.77 | 93 | a-Terpineol |
| Delta-Cadinene | 13.87 | 161 | Linalool |
| 2,4-Nonadienal | 13.99 | 81 | 2-Octenal |
| Citronellol | 13.83 | 69 | Citronellol |
| Nerol | 14.31 | 69 | Nerol |
| 2,4-Nonadienal | 14.51 | 81 | 2-Octenal |
| β-damascone | 14.53 | 177 | β-damascone |
| β-Damascenone | 14.58 | 69 | β-Damascenone |
| Geraniol | 14.81 | 69 | Geraniol |
| Geranylacetone | 14.91 | 69 | Geranylacetone |
| α-ionone | 14.91 | 177 | α-ionone |
| Propanoic acid | 15.22 | 69 | 2-Octenal |
| Cis-Farnesol | 15.69 | 69 | 1-Hexanol |
| β-ionone | 15.83 | 177 | β-ionone |
| Trans-β-ionone-5,6-epoxide | 16.40 | 123 | β-ionone |
| Trans-β-ionone-5,6-epoxide | 16.54 | 123 | β-ionone |
| Pseudo-ionone | 16.87 | 124 | Pseudo-ionone |
| Pseudo-ionone | 17.76 | 124 | Pseudo-ionone |
| Nonanoic acid | 18.01 | 124 | 2-Octenal |
