# Supplementary material for: Field-Grown Grapevine Berries Use Carotenoids and the Associated Xanthophyll Cycles to Acclimate to UV Exposure Differentially in High and Low Light (Shade) Conditions
Source: Front Plant Sci. 2016 Jun 10;7:786. doi: 10.3389/fpls.2016.00786 (PMC4901986; doi:10.3389/fpls.2016.00786)
Supplement: Supplementary file 9 [file Table_3.DOCX]

|  | Development | Exposure | UVB-attenuation | Exposure ×  Development | UVB-attenuation ×  Development | UVB-attenuation × Exposure | UVB-attenuation ×  Exposure ×  Development |
| --- | --- | --- | --- | --- | --- | --- | --- |
| Total chlorophylls | 1205.26 | 0.00 | 0.13 | 22.05 | 4.69 | 0.49 | 8.25 |
| Glucose | 1126.48 | 7.49 | 0.46 | 4.82 | 0.30 | 0.07 | 0.05 |
| Total sugars | 1060.99 | 8.53 | 0.57 | 4.93 | 0.37 | 0.04 | 0.03 |
| Fructose | 1000.92 | 9.63 | 0.69 | 5.02 | 0.45 | 0.02 | 0.02 |
| Total carotenoids | 859.01 | 6.88 | 5.77 | 41.33 | 13.06 | 0.59 | 14.15 |
| Total xanthophylls | 562.22 | 39.86 | 19.80 | 38.81 | 40.66 | 4.12 | 18.56 |
| Total acids | 395.67 | 0.00 | 0.32 | 9.30 | 1.36 | 1.25 | 0.09 |
| Tartaric acid | 329.09 | 15.00 | 0.17 | 1.84 | 0.76 | 0.15 | 0.16 |
| Malic acid | 206.64 | 35.33 | 0.01 | 11.92 | 1.45 | 1.82 | 0.48 |
| Succinic acid | 125.14 | 14.57 | 0.29 | 10.82 | 0.04 | 0.01 | 0.00 |
